# Comprehensive genomic landscape and precision therapeutic approach in biliary tract cancers

**DOI:** 10.1002/ijc.33230

**Published:** 2020-08-28

**Authors:** Ryosuke Okamura, Razelle Kurzrock, Robert J. Mallory, Paul T. Fanta, Adam M. Burgoyne, Bryan M. Clary, Shumei Kato, Jason K. Sicklick

**Affiliations:** ^1^ Center for Personalized Cancer Therapy UC San Diego Moores Cancer Center La Jolla California USA; ^2^ Division of Hematology‐Oncology UC San Diego Moores Cancer Center La Jolla California USA; ^3^ Division of Surgical Oncology, Department of Surgery UC San Diego Moores Cancer Center La Jolla California USA

**Keywords:** biliary tract cancers, biomarker, cholangiocarcinoma, circulating tumor DNA, liquid biopsy, molecular profiling, personalized cancer therapy

## Abstract

Biliary tract cancers have dismal prognoses even when cytotoxic chemotherapy is administered. There is an unmet need to develop precision treatment approaches using comprehensive genomic profiling. A total of 121 patients with biliary tract cancers were analyzed for circulating‐tumor DNA (ctDNA) and/or tissue‐based tumor DNA (tissue‐DNA) using clinical‐grade next‐generation sequencing: 71 patients (59%) had ctDNA; 90 (74%), tissue‐DNA; and 40 (33%), both. Efficacy of targeted therapeutic approaches was assessed based upon ctDNA and tissue‐DNA. At least one characterized alteration was detected in 76% of patients (54/71) for ctDNA [median, 2 (range, 0‐9)] and 100% (90/90) for tissue‐DNA [median, 4 (range, 1‐9)]. Most common alterations occurred in *TP53* (38%), *KRAS* (28%), and *PIK3CA* (14%) for ctDNA vs *TP53* (44%), *CDKN2A*/B (33%) and *KRAS* (29%) for tissue‐DNA. In 40 patients who had both ctDNA and tissue‐DNA sequencing, overall concordance was higher between ctDNA and metastatic site tissue‐DNA than between ctDNA and primary tumor DNA (78% vs 65% for *TP53*, 100% vs 74% for *KRAS* and 100% vs 87% for *PIK3CA* [But not statistical significance]). Among 80 patients who received systemic treatment, the molecularly matched therapeutic regimens based on genomic profiling showed a significantly longer progression‐free survival (hazard ratio [95%confidence interval], 0.60 [0.37‐0.99]. *P* = .047 [multivariate]) and higher disease control rate (61% vs 35%, *P* = .04) than unmatched regimens. Evaluation of ctDNA and tissue‐DNA is feasible in biliary tract cancers.

## Abstract 2

**What's new?**

There is an unmet need for investigating precision therapy approaches in biliary tract cancers using next‐generation sequencing techniques. This study shows that circulating tumour DNA (ctDNA) and tissue DNA tests are complementary, since they often reveal different alterations likely due to tumour heterogeneity. Most biliary tract cancer patients had at least one characterised alteration in ctDNA and in tissue DNA. Among 80 patients who received systemic chemotherapy, molecularly‐matched therapeutic regimens based on genomic profiling were associated with better treatment response and progression‐free survival than unmatched therapies. Evaluation of ctDNA and tissue‐DNA is feasible and potentially beneficial in biliary tract cancers.

**Abbreviations**
CAP: College of American Pathologist
C‐HCC: combined or mixed cholangio‐hepatocellular carcinoma
CI: confidence interval
CLIA: clinical laboratory improvement amendments
CR: complete response
ctDNA: circulating‐tumor DNA
ECOG‐PS: Eastern Cooperative Oncology Group Performance Status
EHCC: extrahepatic cholangiocarcinoma)
GBCA: gallbladder cancer
GEMOX: gemcitabine plus oxaliplatin
HR: hazard ratio
IHC: immunohistochemistry
IHCC: intrahepatic cholangiocarcinoma
MSI: micro microsatellite instability
NGS: next‐generation sequencing
OS: overall survival
PD: progressive disease
PD‐L1: programmed death‐ligand 1
PFS: progression‐free survival
PR: partial response
RECIST: Response Evaluation Criteria in Solid Tumors
SD: stable disease
TMB: tumor mutational burden

## 1 INTRODUCTION

Despite their low incidence, the mortality from biliary tract cancers is high. Biliary tract cancers are generally categorized as intrahepatic cholangiocarcinoma (IHCC), extrahepatic cholangiocarcinoma (EHCC) and gallbladder cancer (GBCA). Also, combined or mixed cholangio‐hepatocellular carcinoma (C‐HCC) which comprises histopathological features of cholangiocarcinoma and hepatocellular carcinoma is occasionally seen. Traditionally, systemic therapy approaches have been the same for all of these tumors, regardless of the tumor type as they were assumed to have similar biologies.^1^, ^2^

Biliary tract cancers mostly present with locally advanced disease or metastatic lesions precluding surgical resection. Moreover, they all have poor prognoses even when systemic chemotherapy is administered. In several clinical trials, the median progression‐free survival (PFS) and median overall survival (OS) of multi‐agent regimens in the advanced settings remain dismal despite being limited to patients with good performance status and without hyperbilirubinemia (median PFS: 5.8‐8.0 months for gemcitabine plus cisplatin, 4.2‐5.7 months for gemcitabine plus oxaliplatin [GEMOX], 5.8 months for GEMOX with erlotinib and 11.8 months for gemcitabine cisplatin plus nab‐paclitaxel; median OS: 11.2‐11.7 months for gemcitabine plus cisplatin, 9.5‐15.4 months for GEMOX, 9.5 months for GEMOX with erlotinib and 19.2 months for gemcitabine cisplatin plus nab‐paclitaxel).^3^, ^4^, ^5^, ^6^, ^7^Thus, the goals of chemotherapy in advanced biliary tract cancer patients are mostly palliative in nature.^1^ As a result, personalized, molecular targeted approaches have emerged as a potential approach for treating malignancies with high mortality.^8^, ^9^ In a meta‐analysis of 32 149 patients with diverse cancers who underwent early phase clinical trials, targeted therapy approaches without specific biomarkers had significantly worse clinical outcomes (ie, objective response rate, PFS and OS) when compared to patients who received targeted therapies based upon biomarkers.^10^ However, previous clinical trials that utilized targeted therapies in biliary tract cancers have not shown clinically significant improvements in overall response rates so far (eg, 31‐33% for GEMOX or gemcitabine/irinotecan with panitumumab targeting EGFR among *KRAS* wild‐type biliary tract cancers; and 15% for an FGFR inhibitor among FGFR‐altered cholangiocarcinoma).^11^, ^12^, ^13^ Some of the limitations to the previous targeted therapy approaches may be due to spatial or temporal tumor heterogeneity that may lead to the lack of response with single targeted approaches.^14^ Also, tissue biopsies of biliary tract cancers can be challenging to safely obtain with adequate tissue quality for comprehensive molecular testing. Thus, the blood‐derived circulating tumor DNA (ctDNA) technique has some advantages over tissue‐DNA sequencing since it is less‐invasive and potentially enables real‐time monitoring of genomic evolution. Herein, we assessed the genomic landscape of ctDNA along with tissue‐DNA using clinical‐grade next‐generation sequencing (NGS), as well as also investigated the efficacy using genomic profiling data from both approaches to administer molecularly matched targeted therapies to patients with biliary tract cancers.

## 2 METHODS

### 2.1 Patients

We collected the genomic and clinical data of patients pathologically diagnosed as IHCC, EHCC, GBCA or C‐HCC, who were presented to the UC San Diego Moores Cancer Center between March 2012 and March 2019. The study was conducted consistent with the IRB‐approved protocol *Profile Related Evidence Determining Individualized Cancer Therapy* (UCSD‐PREDICT study: NCT02478931) parameters and any investigational therapies for which the patients gave consent. All investigations were performed in accordance with the guidelines of the UC San Diego Internal Review Board and the Declaration of Helsinki.

### 2.2 Clinical grade next‐generation sequencing

#### 2.2.1 Blood‐derived circulating tumor DNA

ctDNA assay for all blood samples was performed by a clinical laboratory improvement amendments (CLIA) licensed and College of American Pathologist (CAP) accredited clinical laboratory, *Guardant Health*, *Inc*. (Redwood City, California; http://www.guardanthealth.com; panels of 68‐73 genes; Table S1) and sequenced cancer‐associated genes to identify somatic alterations with high analytic sensitivity and high specificity, as previously described.^15^ In this study, only characterized genomic alterations were used for analysis (synonymous alterations or variants of unknown significance were excluded).

#### 2.2.2 Tumor tissue‐DNA

Tissue‐DNA assay for all tumor samples was performed by a CLIA‐licensed CAP‐accredited laboratory, *Foundation Medicine*, *Inc*. (Cambridge, Massachusetts; https://www.foundationmedicine.com; panels of 236‐324 genes; Table S2). Also in tissue‐DNA, only characterized alterations were analyzed. The sequencing was designed to include all genes somatically altered in human solid malignancies that were validated as targets for therapy, either approved or in clinical trials, and/or that were unambiguous oncogenic drivers based on available recent knowledge.^16^ Micro microsatellite instability (MSI) and tumor mutational burden (TMB) were also evaluated in tumor tissues as the biomarkers which have entered clinical practice for immunotherapies.^17^, ^18^, ^19^, ^20^

### 2.3 Definition and statistical analysis

In this series, hilar cholangiocarcinoma was classified as EHCC. Genomic concordance between ctDNA and tissue‐DNA tests was assessed in the three most commonly altered genes in ctDNA at the gene level and described with overall concordance rate. The Kappa values were interpreted by commonly used agreement categories: from 1 (perfect agreement) to 0 (no agreement, the same as would be expected by chance). When patients were stratified according to tissue biopsy site and time interval between blood draw and tissue biopsy, the difference in concordance rate was compared by Fisher's exact test. All statistical analysis was done using SPSS Statistics version 24 software (IBM Corporation, Armonk, New York).

#### 2.3.1 Matched targeted therapy based on molecular profiling

We assessed the efficacy of precision oncology approaches based on ctDNA and/or tissue‐DNA molecular profiling. For this analysis, treatment regimens that were initiated after the dates of blood draw for ctDNA analysis and tissue biopsy were only studied (the first regimen initiated after molecular profiling for each patient). When at least one drug was administered and it targeted at least one genomic alteration in either ctDNA or tissue‐DNA or both, treatment was considered “matched therapy” as previously described.^8^ We also considered checkpoint inhibitors matched to mismatch‐repair gene alteration (eg, alteration in *MLH1*, *MSH2*), programmed death‐ligand 1 (PD‐L1) immunohistochemistry (IHC), or high/intermediate tumor mutational burden (TMB: high [≥20 mutations/mb]; and intermediate [6‐19 mutations/mb]) and certain alterations (including but not limited to *PDL1* amplification) as “matched therapy”. In addition, even when treated with a conventional platinum‐based regimen (eg, cisplatin plus gemcitabine), the patient was considered “matched” if the genomic profiling includes at least one *BRCA*‐associated gene alteration (eg, *BRCA2*, *BAP1*, *ATM*). Tumor response was assessed by means of computed tomography or magnetic resonance imaging at every 8 to 12 weeks, using modified Response Evaluation Criteria in Solid Tumors (RECIST) 1.1 evaluation: complete response (CR), partial response (PR); stable disease (SD); and progressive disease (PD).^21^ PFS was defined as the time from the initiation of the regimen to progressive disease (PD) or all cause death (counted as censored if a patient still survives without progression on the date of data cutoff [April 2019] or if the regimen was switched to another regimen without PD on imaging [eg, due to toxicity or patient's preference]). The sample size was mainly determined by the number of patients for whom data were available among the patients who were consented to the UCSD‐PREDICT study (ClinicalTrials.gov, NCT02478931).

## 3 RESULTS

### 3.1 Patient demographics and genomic landscape in next‐generation sequencing

A total of 121 patients with biliary tract cancers were evaluated: 40 patients (33%) had both ctDNA and tissue‐DNA analyses, 31 (26%) had only ctDNA analysis and 50 (41%) had only tissue‐DNA analysis (Figure S1). Fifty one percent of the 112 patients were male, and the median age at disease diagnosis was 63 years (Table 1). Tumor type was IHCC in 49% (n = 59), EHCC in 22% (n = 26), GBCA in 24% (n = 29) and C‐HCC in 5.8% (n = 7), respectively. Median follow‐up time from disease diagnosis was 27.8 months (95% confidence interval [CI], 23.4‐32.2).

**TABLE 1:** Clinical characteristics of biliary tract cancer patients (n = 121)

*Basic characteristics (all patients*, *n = 121)*	n (%)
Median age at diagnosis (range) (years)	62.6 (31.2‐88.5)
Sex	
Male	62 (51.2%)
Female	59 (48.8%)
Ethnicity	
Caucasian	67 (55.4%)
Hispanic	32 (26.4%)
Asian	11 (9.1%)
Other/unknown	11 (9.1%)
Tumor type	
Intrahepatic cholangiocarcinoma (IHCC)	59 (48.8%)
Extrahepatic cholangiocarcinoma (EHCC)	26 (21.5%)
Gallbladder carcinoma (GBCA)	29 (24.0%)
Cholangio‐hepatocellular carcinoma (C‐HCC)	7 (5.8%)
*Patients who had ctDNA analysis (n = 71)*	n (%)
Disease status at the time of blood draw for ctDNA	
Metastatic, locally advanced, or recurrent disease	67 (94.4%)
Surgically resectable (blood was biopsied postoperatively)^a^	4 (5.6%)
Number of patients with ≥1 characterized alteration	54 (76.1%)
Median number of characterized alterations per patient (range)	2 (0–9)
IHCC (n = 36)	2 (0‐9)
EHCC (n = 19)	1 (0‐6)
GBCA (n = 13)	2 (0‐7)
C‐HCC (n = 3)	0 (0‐2)
Median of total %ctDNA per patient (%)	1.1 (0.0‐119.7)
*Patients who had tissue‐DNA analysis (n = 90)*	n (%)
Disease status at the time of tissue biopsy for tissue‐DNA	
Metastatic, locally advanced, or recurrent disease	73 (81.1%)
Surgically resectable	17 (18.9%)
Biopsy site	
Primary tumor	70 (77.8%)
Metastatic sites	20 (22.2%)
Number of patients with ≥1 characterized alteration	90 (100%)
Median number of characterized alterations per patient (range)	4 (1‐9)
IHCC (n = 41)	3 (1‐8)
EHCC (n = 20)	4 (1‐7)
GBCA (n = 24)	4.5 (1‐9)
C‐HCC (n = 5)	4 (2‐6)

Abbreviations: ctDNA, circulating‐tumor DNA; C‐HCC, cholangio‐hepatocellular carcinoma; CI, confidence interval; EHCC, extrahepatic cholangiocarcinoma; GBCA, gallbladder carcinoma; IHCC, intrahepatic cholangiocarcinoma.

^a^ Blood draw was performed after radical surgery in three GBCA patients (ID#28, 44 and 91) and stereotactic radiosurgery in one IHCC patient (ID#111).

#### 3.1.1 ctDNA NGS in biliary tract cancer patients (n = 71)

**FIGURE 1:**
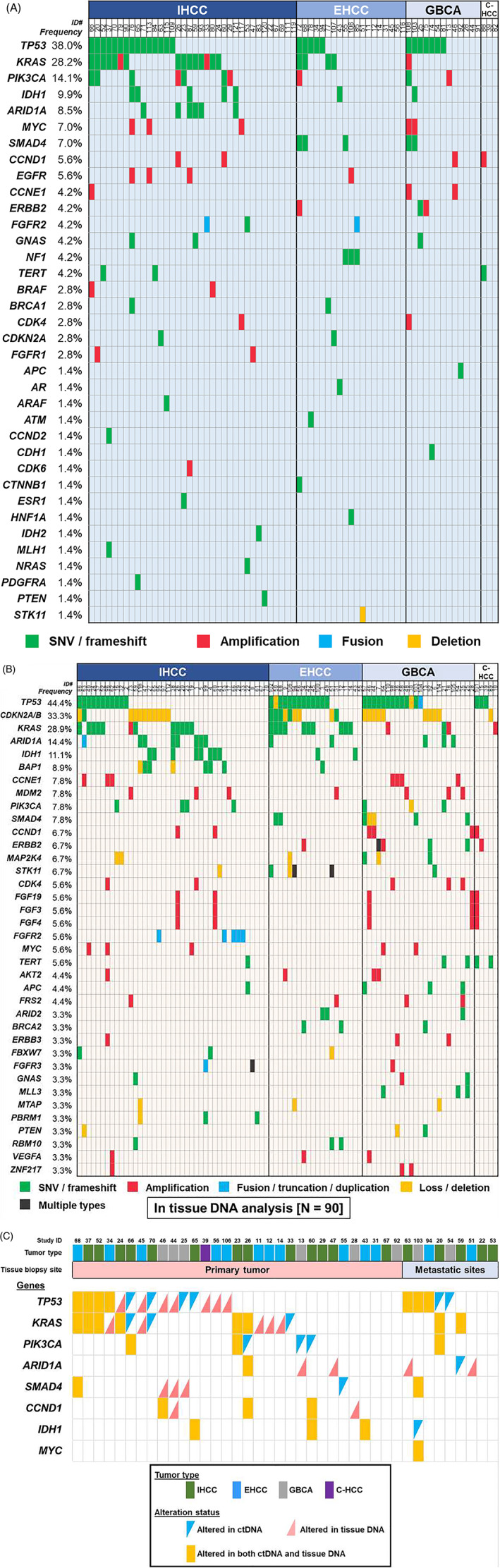
Genomic characterized alterations among patients with biliary tract cancers. A, Frequency of genomic alterations in ctDNA sequencing (n = 71). B, Frequency of genomic alterations in tissue‐DNA sequencing (genes altered in ≥3 patients were only shown; n = 90). C, Association between ctDNA and tissue‐DNA in commonly altered genes among patients whose ctDNA and tissue‐DNA were both analyzed (n = 40)

The ctDNA analyses were performed in advanced disease setting (metastatic, locally advanced or recurrent disease), except for three GBCA cases and one IHCC case whose ctDNA were analyzed postoperatively (Table 1). Of the 71 patients with ctDNA analysis, 76% (n = 54) had at least one characterized alteration in ctDNA. The median number of characterized alterations per patient was 2 (range, 0‐9), and a total of 147 characterized alterations were observed, including 112 mutations (76%), 32 gene amplifications (22%), 2 gene fusions (1.4%) and 1 indel (0.7%). These characterized alterations involved 36 unique genes and included 97 distinct alterations (Figure 1A). The most common genes altered in ctDNA were *TP53* (38%, n = 27), followed by *KRAS* (28%, n = 20) and *PIK3CA* (14%, n = 10). Overall, 85% of these characterized alterations (125 of the 147 alterations) were theoretically targetable with FDA‐approved agents (on‐ or off‐label use; Table S3). In other words, 75% of the patients (n = 53) had at least one characterized alteration targetable with FDA‐approved agents (on‐ or off‐label). Only two patients harbored molecularly identical portfolios (*PIK3CA* amplification) in ctDNA.

#### 3.1.2 Tissue‐DNA NGS in biliary tract cancer patients (n = 90)

Seventy eight percent of the tissue‐DNA analyses (n = 70 of 90) used primary tumor samples while the remaining 22% (n = 20) utilized biopsies from metastatic sites (Table 1). Interestingly, all 90 patients had at least one characterized alteration in the tissue‐DNA (median number of characterized alterations per patient [range], 4 [1–9]). A total of 362 characterized alterations were observed in tissue‐DNA, including 190 mutations (53%), 105 gene amplifications (29%), 52 allelic loss/deletions (14%) and 15 gene fusions/truncations/duplications (4%), which involved 106 different genes and 236 distinct alterations (genes altered in ≥3 samples were shown in Figure 1B). The most common genes altered in tissue‐DNA were *TP53* (44%, n = 40), followed by *CDKN2A/B* (33%, n = 30) and *KRAS* (29%, n = 26). Of the 362 characterized tissue‐DNA alterations, 70% of alterations (252/362) were theoretically targetable with FDA‐approved agent while 96% of the patients (n = 86) had at least one tissue‐DNA characterized alteration which was pharmacologically targetable with FDA‐approved agents. No two patients had molecularly identical tissue‐DNA portfolios.

### 3.2 Genomic concordance between ctDNA and tissue‐DNA sequencing (n = 40)

Overall, 40 patients had both ctDNA and tissue‐DNA NGS. When comparing *TP53*, *KRAS* and *PIK3CA* genes, the overall concordance rate between ctDNA and tissue‐DNA was 68%, 80% and 90%, respectively (Kappa values ranged 0.27‐0.55; Table 2). When comparing according to tissue biopsy site, ctDNA alteration was numerically more concordant with metastatic site DNA than primary tumor DNA in these three genes (overall concordance [Kappa], 78% vs 65% [0.57 vs 0.17] for *TP53*; 100% vs 74% [1.00 vs 0.41] for *KRAS*; and 100% vs 87% [1.00 vs 0.45] for *PIK3CA*). But there were no statistical differences observed (the *P* values ranged .16‐.69; Table 2 and Figure 1C). In terms of temporal effects in the genomic concordance, no clear differences were observed for samples from ≤6 months vs >6 months apart (ie, between blood draw for ctDNA and tissue biopsy) in these genes although the Kappa values were likely higher in the ≤6 months group (74% vs 54% [0.40 vs 0.03] for *TP53*; 82% vs 77% [0.60 vs 0.32] for *KRAS*; and 100% vs 87% [0.61 vs 0.00] for *PIK3CA*; *P* values ranged .28‐.99]).

**TABLE 2:** Concordance of common genes between ctDNA and tissue‐DNA among patients with biliary tract cancers whose ctDNA and tissue‐DNA were both analyzed (n = 40)

*Patients who had both ctDNA and tissue‐DNA sequencing (n = 40)*											
				Tissue‐DNA (+)	Tissue‐DNA (−)				Overall concordance	Kappa^a^ (SE)	
*TP53*	ctDNA (+)			7	6				67.5%	0.27 (0.16)	
	ctDNA (−)			7	20						
*KRAS*	ctDNA (+)			8	3				80.0%	0.53 (0.15)	
	ctDNA (−)			5	24						
*PIK3CA*	ctDNA (+)			3	4				90.0%	0.55 (0.19)	
	ctDNA (−)			0	33						
*Concordance depending on whether primary tumor or metastatic site was biopsied*											
	Primary tumor (n = 31)					Metastatic sites (n = 9)					*P* value (primary tumor vs metastatic sites)
	(+/+)	(+/−, −/+)	(−/−)	Overall concordance	Kappa (SE)	(+/+)	(+/−, −/+)	(−/−)	Overall concordance	Kappa (SE)	
*TP53*	n = 4	n = 11	n = 16	64.5%	0.17 (0.18)	n = 3	n = 2	n = 4	77.8%	0.57 (0.24)	.69
*KRAS*	n = 6	n = 8	n = 17	74.2%	0.41 (0.17)	n = 2	n = 0	n = 7	100%	1.00 (0.00)	.16
*PIK3CA*	n = 2	n = 4	n = 25	87.1%	0.45 (0.22)	n = 1	n = 0	n = 8	100%	1.00 (0.00)	.56
*Concordance based on time interval between blood draw and tissue biopsy*											
	≤6 months (n = 27)					>6 months (n = 13)					*P* value (≤6 vs > 6 months)
	(+/+)	(+/−, −/+)	(−/−)	Overall concordance	Kappa (SE)	(+/+)	(+/−, −/+)	(−/−)	Overall concordance	Kappa (SE)	
*TP53*	n = 5	n = 7	n = 15	74.1%	0.40 (0.19)	n = 2	n = 6	n = 5	53.8%	0.03 (0.28)	.28
*KRAS*	n = 7	n = 5	n = 15	81.5%	0.60 (0.16)	n = 1	n = 3	n = 9	76.9%	0.32 (0.25)	>.99
*PIK3CA*	n = 3	n = 3	n = 21	88.9%	0.61 (0.20)	n = 0	n = 1	n = 12	92.3%	0.00 (0.00)	>.99

*Note*: Most common three genes in ctDNA sequencing were assessed at the gene level. *P* values in bold are those less than 0.05.

Abbreviation: ctDNA, circulating‐tumor DNA.

^a^ The closer the Kappa to 1, the more the concordance.

### 3.3 Treatment outcome of personalized matched therapy approaches in advanced biliary tract cancers (n = 80)

**FIGURE 2:**
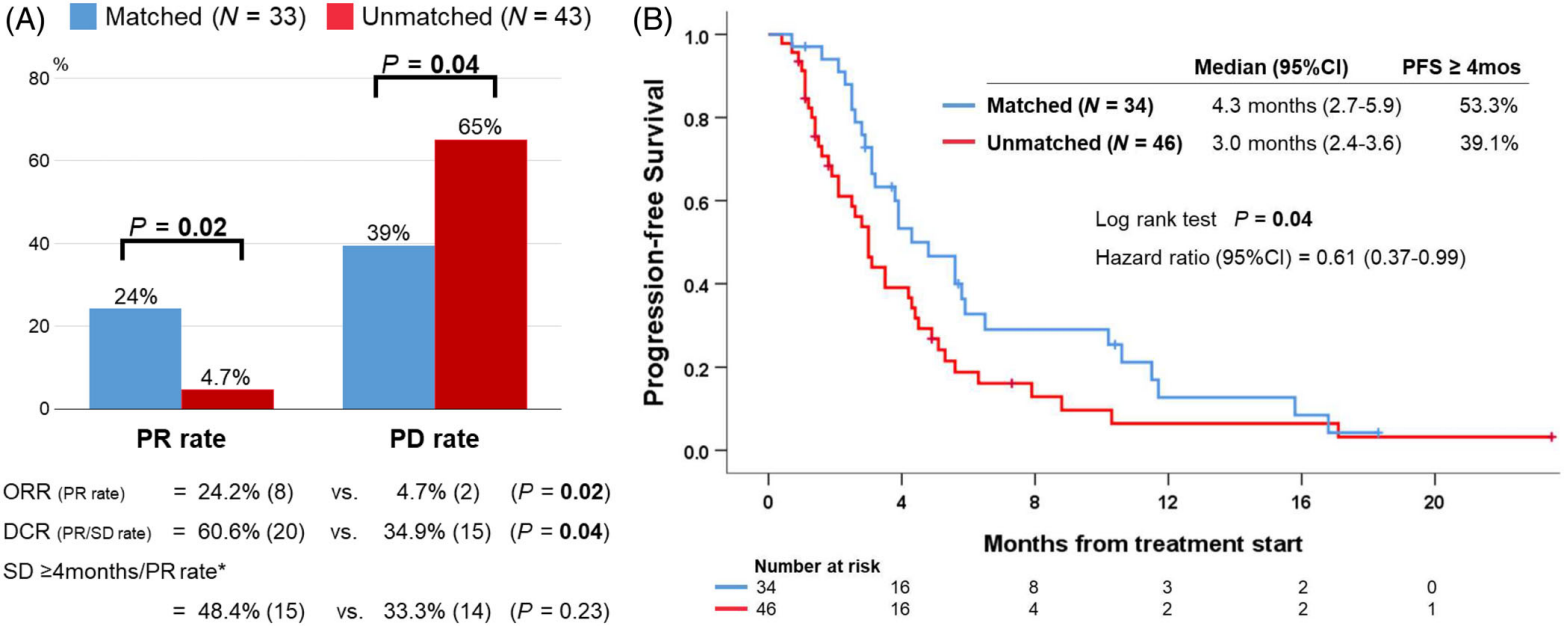
Comparisons of treatment outcome between matched regimens and unmatched regimens among patients who received systemic therapies after molecular profiling. A, Best response during the treatment (76 of 80 patients [95%] were available for RECIST evaluation). Among 76 evaluable patients, PR was observed in 10 patients (13%), SD in 25 (33%) and PD in 41 (54%) as the best response during the therapies after their molecular profiling (no one had complete response from the treatments). B, Progression‐free survival (n = 80)

Among the 121 patients with biliary tract cancers, 80 patients had systemic therapies initiated after the molecular profiling in locally advanced or metastatic disease setting (adjuvant intent chemotherapy was excluded) (Figure S1). Of these 80 treated patients, 43% (n = 34) were administered at least one drug matched to their profiling results (detailed genomic information was shown in Table S4). The matched targeted therapies include molecular targeting therapies for genomic alterations in ctDNA and/or tissue‐DNA (n = 29), immunotherapies for PD‐L1 IHC status (n = 3) or mismatch repair deficiency (n = 1) and a combination of molecular targeting therapy with immunotherapy for TMB status (n = 1). These matched patients received a median of two drugs (range, 1‐3), and the regimens were administered as their first‐line treatments in 67% of the patients (n = 23). Eleven patients (32%) were treated with gemcitabine with platinum agents and their tissue‐DNA included at least one alteration in *BRCA*‐associated genes (ie, alterations in *ATM*, *BAP1*, *BRCA2*, *CHEK2*, *FANCL* or *RAD50* gene). Patients with *FGFR* fusion (ID#33) and *IDH1* alteration (ID#38) received anti‐FGFR and IDH therapies, respectively. On the other hand, the remaining 46 of the 80 patients (57%) were treated with unmatched regimens, which mostly used gemcitabine‐based regimens (gemcitabine with platinums [n = 22]; gemcitabine with capecitabine [n = 6]; gemcitabine monotherapy [n = 9]) and other regimens (n = 9). Additionally, 87% (n = 40) of the unmatched patients were treated with these regimens as their first‐line treatments. For instance, 31% of the treated patients (n = 25/80) harbored *KRAS* alterations in either of the tissue DNA or ctDNA testing or both (n = 14, only in tissue; n = 6, only in ctDNA; and n = 5, in both), and five patients of them (ID#23, #37, #38, #52 and #59) received matched treatment regimens including trametinib, a MEK inhibitor (Table S4). Also, five patients (ID#12, #33, #34, #66 and #86) received other matched regimens based on their tissue DNA and/or ctDNA testing (eg, a FGFR inhibitor for *FGFR2* fusion [ID#33]). The remaining 15 patients mostly received gemcitabine‐based unmatched regimens. The matched and unmatched patients were similar in regard to key basic characteristics such as pretreatment physical conditions (age, ECOG‐PS, or total bilirubin level), tumor site or extent of disease (Table S5). RECIST evaluation was available in 76 of the 80 treated patients (95%), and the PR rate was significantly higher in the matched regimen group vs the unmatched regimen group (24% [n = 8 of 33] vs 4.7% [n = 2 of 43], *P* = .02) while the PD rate was significantly lower in the matched regimen group (39% [n = 13 of 33] vs 65% [n = 28 of 43], *P* = .04; Figure 2A). Consistent with the response analysis, Kaplan‐Meier curves showed that the matched regimen group had a significantly longer PFS time than the unmatched regimen group (median PFS time, 4.3 vs 3.0 months, *P* = .04; Figure 2B). Importantly, the matched regimens remained significantly associated with better PFS even when age, sex, performance status, tumor type, extent of disease, presence of prior radical surgery, number of prior regimens and the number of drugs administered were considered as confounding factors in the multivariate analysis (HR [95%CI], 0.60 [0.37‐0.99]; *P* = .047; Table 3).

**TABLE 3:** Exploration of prognostic factors for progression‐free survival among the treated patients with biliary tract cancers (n = 80)

Characteristics	Univariate		Multivariate^a^	
	Median PFS (months)	*P* value	HR (95%CI)	*P* value
Age, years^b^				
≥63 (n = 37) vs <63 (n = 43)	3.5 vs 3.9	.54	—	—
Sex				
Men (n = 41) vs Women (n = 39)	3.0 vs 4.8	.22	—	—
ECOG‐PS				
2‐3 (n = 21) vs 0‐1 (n = 59)	3.9 vs 3.2	.70	—	—
Total bilirubin, mg/dL^c^				
>3.6 (n = 8) vs ≤3.6 (n = 72)	2.8 vs 3.5	.26	—	—
Tumor type				
IHCC (n = 41) vs not (n = 39)	3.5 vs 3.8	.25	—	—
Extent of disease				
Metastatic (n = 67) vs locally advanced (n = 13)	3.2 vs 5.1	.39	—	—
Extent to extrahepatic (n = 63) vs not (n = 17)	3.2 vs 5.1	.48	—	—
Lung metastasis (n = 17) vs not (n = 63)	2.8 vs 3.8	.12	1.35 (0.76‐2.41)	.31
Peritoneal metastasis (n = 26) vs not (n = 54)	3.1 vs 4.2	.38	—	—
Radical surgery prior to chemotherapy				
Yes (n = 30) vs no (n = 50)	3.5 vs 3.8	.14	0.68 (0.40‐1.15)	.15
Treatment				
Matched (n = 34) vs unmatched (n = 46)	4.3 vs 3.0	.**04**	0.60 (0.37‐0.99)	.**047**
Administered as first line (n = 62) vs ≥second line (n = 18)	3.5 vs 3.9	.78	—	—
Single drug (n = 19) vs ≥2 drugs (n = 61)	2.8 vs 3.9	.22	—	—

*Note*: *P* values in bold are those less than 0.05.

Abbreviations: CI, confidence interval; ECOG‐PS, Eastern Cooperative Oncology Group Performance Status; HR, hazard ratio; IHCC, intrahepatic cholangiocarcinoma; PFS, progression‐free survival.

^a^ Variables with ≤0.15 in the univariate analysis were included in the multivariate analysis.

^b^ Age at diagnosis. Dichotomized by the median.

^c^ Total bilirubin at the time of treatment start. Dichotomized by (3 × institutional upper limit of normal [1.2 mg/dL]).

## 4 DISCUSSION

Most biliary tract cancers are unresectable at presentation and often have metastases to intrahepatic sites, lymph nodes or the peritoneum.^22^ Even in surgically resectable cases, involvement of surgical margin often occur and is associated with high rates of disease recurrence.^23^ At present, gemcitabine‐based combination regimens are globally accepted as the systemic chemotherapy regimen for advanced biliary tract cancer patients. However, the prognosis remains poor.^3^, ^4^, ^5^, ^6^, ^7^ Thus, there is an unmet need for novel therapeutic approaches for these cancers. Precision oncology approaches have recently shown promising responses in diverse cancer types.^7^, ^8^, ^9^, ^10^, ^24^ To the best of our knowledge, the detailed comprehensive genomic landscape of biliary tract cancers by using clinical‐grade ctDNA as well as its concordance analysis with tissue‐DNA are limited.^25^ We now demonstrate that each biliary tract cancer patient has distinct pattern of ctDNA and tissue‐DNA genomic alterations which are frequently druggable and that targeted matched therapies based on the molecular profiling are associated with higher response rates and longer progression‐free survival.

Interestingly, 76% of the patients had at least one characterized ctDNA alteration while 100% had at least one characterized alteration found in tissue‐DNA. In addition, the median number of alterations per patient (range) was two (0‐9) in ctDNA and four (1‐9) in tissue‐DNA. The most common alterations occur in *TP53* (38%), *KRAS* (28%) and *PIK3CA* (14%) for ctDNA while in *TP53* (44%), *CDKN2A/B* (33%) and *KRAS* (29%) for tissue‐DNA, respectively. The frequencies in both ctDNA and tissue‐DNA NGS were consistent with previous reports.^26^, ^27^ In the majority of the commonly altered genes in this series, such as *TP53*, *KRAS*, *ARID1A* and *IDH*, similar percentages were seen between ctDNA and tissue‐DNA. However, *CDKN2A*/B alterations were detected in only 3% of patients by ctDNA, as compared to 33% by tissue‐DNA NGS. This discrepancy might be attributable to the possibility that earlier versions of the ctDNA panel did not capture allelic loss in this gene. Moreover, among 40 patients who had both ctDNA and tissue‐DNA NGS analyses, no two patients had identical results from these molecular profiling. Moreover, no IHCC patient had characterized *SMAD4* or *ERBB2* alterations in either ctDNA or tissue‐DNA NGS in this series. In terms of druggability, 75% of patients who had ctDNA analysis and 96% of patients whose tissue‐DNA were analyzed had at least one alteration that was theoretically targetable with FDA‐approved drugs (on‐ or off‐label), respectively. A previous study reported that 55% of patients with biliary tract cancers had therapeutically relevant characterized ctDNA alterations although genes considered as targetable were somewhat different with our study.^25^ Altogether, nearly all biliary tract cancer patients had unique pattern of genomic alterations in ctDNA and tissue‐DNA NGS, indicating that these two different sequencing techniques can be complementary. These findings also suggest the potential for precision clinical trials in biliary tract cancers, as well as that customized molecular targeting based on each individual genomic portfolio may be necessary to improve outcomes.^8^, ^28^, ^29^, ^30^

Overall concordance between ctDNA and tissue‐DNA was 68% to 90% for *TP53*, *KRAS* and *PIK3CA* genes. Discrepancies between the two tests may be due to the intertumor and intratumor genetic heterogeneity.^14^ Also, cases with positive in ctDNA and negative in tissue‐DNA for certain genes, such as *TP53* or *KRAS*, may be explained by age‐related or therapy‐related clonal hematopoiesis.^31^, ^32^ When stratified according to tissue biopsy sites, ctDNA was numerically more concordant with metastatic site tissue‐DNA than primary tumor DNA, although there were no statistical differences observed (78% vs 65% in *TP53*, 100% vs 74% in *KRAS* and 100% vs 87% in *PIK3CA*). This finding likely suggests that additional mutations develop in metastatic tumors and that ctDNA may be able to detect DNA shed into bloodstream from the tumors throughout the patient's whole body.^14^, ^33^ Also, biomarker profiling of either metastatic site tissue‐DNA or ctDNA may be more effective in selection of therapy than interrogating primary tumor sites. Moreover, there was a temporal effect on concordance with shorter time apart between blood draw and tissue biopsy (≤6 months): higher concordances in *TP53* and *KRAS* and greater Kappa values in *TP53*, *KRAS* and *PIK3CA* than >6 months. However, further studies with larger sample sizes are required for validation.

Given the emerging role of precision matched therapies, we assessed the efficacy of molecularly matching drugs in patients with biliary tract cancers.^8^, ^9^ Importantly, the matched targeted regimens had a higher response rate and longer PFS (PR rate, 24% vs 4.7%, *P* = .02; median PFS time, 4.3 vs 3.0 months, *P* = .04) than regimens unmatched to NGS results. It should be noted that about half of unmatched regimens were gemcitabine with platinum agents, which are commonly used as the first line for biliary tract cancers, but the median PFS time was 3 months in current study which is shorter compared to previous reports.^3^, ^6^ This discrepancy may result from the issue that our study was not performed in prospective exploratory setting such as randomized control trials, but in more pragmatic setting including patients with history of previous aggressive therapy (23%), poor performance status (ECOG‐PS 2‐3, 26%) or abnormal total bilirubin levels (> the institutional upper limit of normal [1.2 mg/dL], 23%). On the other hand, 11 patients were treated with gemcitabine plus platinum regimens in the setting of ≥1 characterized alterations in *BRCA*‐associated genes that were considered as the molecularly matched group. Previous studies have suggested that similar to ovarian and breast cancers, cholangiocarcinoma harboring *BRCA*‐associated gene alterations are more sensitive to platinum‐based therapy.^34^, ^35^, ^36^ In fact, when we investigated the patients treated with gemcitabine and platinums in this series, the matched group tended to have a longer median PFS, although there was no statistical significance (5.8 vs 3.1 months; Figure S2). We also assessed the treatment outcomes according to the matching score (the number of targeted gene alterations divided by the total number of alterations observed in NGS; unmatched patients had a score of 0%) as reported previously.^8^, ^28^, ^37^ However, in this series, the number of patients with higher matching score (>50%) was small (13%). Thus, the matching score failed to discriminate the treatment response and PFS with statistical differences (Figure S3). In terms of the efficacy of the matched therapy approaches in patients’ overall survival, the matched patients had a longer median OS time (11.9 vs 7.9 months) and 12‐month‐OS rate (47% vs 39%) over the unmatched patients, although these were not statistical significant (Figure S4). Among treated patients, the unmatched regimens were more often administered as a first line setting than the patients with matched regimens and had a shorter interval between advanced disease diagnoses to initiation of the treatment (Table S5). Therefore, the introduction of matched regimens in earlier phase of disease may require in the future. Historically, several molecularly targeted trials in biliary tract cancers have failed to show favorable outcomes.^38^ Negative studies to date may be due to the lack of biomarker selection or the existence of oncogenic co‐alterations. Thus, individually customized targeted therapy regimens may be required to improve clinical outcomes of patients with advanced biliary tract cancers.

The current study has several limitations. First, not all patients had both ctDNA and tissue‐DNA NGS results and there can be discordance between ctDNA and tissue DNA results. Further studies utilizing both ctDNA and tissue‐DNA NGS are warranted. Also, our classification of matched or unmatched treatment may not be accurate due to the lack of either ctDNA or tissue‐DNA NGS, whereby some unmatched patients may actually have received an unrecognized molecularly matched therapy. In addition, the small number of patients precludes further investigation of the concordance between ctDNA and tissue‐DNA. Additional studies with larger sample sizes are needed. Finally, it is possible that other unmeasurable, or unknown, but important confounding factors were not considered in comparison of treatment strategies. For these issues, randomized controlled trials may be more ideal.

In conclusion, our study demonstrates that biliary tract cancer patients mostly had at least one characterized alteration in ctDNA (76% of blood samples) and tissue‐DNA (100% of tissue samples). Concordance was higher between ctDNA and metastatic site tissue‐DNA than between ctDNA and primary tumor DNA. Among patients who received chemotherapy after the genomic profiling, molecularly matched regimens based on biomarkers showed a statistically longer PFS and higher disease control rate over unmatched regimens. Further investigations of biomarker‐driven therapies using clinical‐grade NGS in blood and tissue are warranted for developing new and better treatment strategies for patient with biliary tract cancer.

## CONFLICT OF INTEREST

Dr Razelle Kurzrock receives research funding from Genentech, Merck Serono, Pfizer, Boehringer Ingelheim, TopAlliance, Takeda, Incyte, Debiopharm, Medimmune, Sequenom, Foundation Medicine, Konica Minolta, Grifols, Omniseq and Guardant, as well as consultant and/or speaker fees and/or advisory board for X‐Biotech, Loxo, Neomed, Pfizer, Actuate Therapeutics and Roche. She has an equity interest in IDbyDNA and CureMatch Inc and serves on the Board of CureMatch and CureMetrix. Shumei Kato received research funds from ACT Genomics, Sysmex, Konica Minolta and OmniSeq, as well as consultant fees for Foundation Medicine. He received speaker fees from Roche. Jason Sicklick received research funds from Foundation Medicine Inc., Novartis Pharmaceuticals and Amgen, as well as consultant fees from Loxo, Grand Rounds and Deciphera. He received speaker fees from Roche. Adam Burgoyne has served as a consultant for Genentech, Deciphera, Exelixis and Eisai. All other authors have no relationships to disclose.

5

## ETHICS STATEMENT

Our study was approved by UC San Diego Institutional Review Board. Written signed informed consent was obtained from all participants.

## Supporting information


**Appendix S1**: Supporting information

## Data Availability

The data that support the findings of our study are available upon request from the corresponding author. The data are not publicly available due to privacy or ethical restrictions.
