# Supplementary material for: Comprehensive genomic landscape and precision therapeutic approach in biliary tract cancers
Source: Int J Cancer. 2020 Aug 28;148(3):702–12. doi: 10.1002/ijc.33230 (PMC7739197; doi:10.1002/ijc.33230)
Supplement: Supplementary file 1 — Appendix S1: Supporting information [file IJC-148-702-s001.pdf]

Comprehensive Genomic Landscape and Precision Therapeutic Approach in Biliary Tract Cancers

Ryosuke Okamura, Razelle Kurzrock, Robert J. Mallory, Paul T. Fanta,
Adam M. Burgoyne, Bryan M. Clary, Shumei Kato, and Jason K. Sicklick

CONTENTS

- **Supplementary Tables.**
- **Supplementary Figures.**

Supplementary Table 1. Next-generation sequencing gene panels for blood-derived ctDNA (*Guardant, Inc.*) (N=71).
Panel 1a. 73 gene panel of ctDNA (N=41 blood samples).

**POINT MUTATIONS**				**AMPLIFICATIONS**	**FUSIONS**	**INDELS**	
AKT1	*ALK*	*APC*	*AR*	*AR*	*ALK*	*APC*	*ARID1A*
ARAF	*ARID1A*	*ATM*	*BRAF*	*BRAF*	*FGFR2*	*ATM*	*BRCA1*
BRCA1	*BRCA2*	*CCND1*	*CCND2*	*CCND1*	*FGFR3*	*BRCA2*	*CDH1*
CCNE1	*CDH1*	*CDK4*	*CDK6*	*CCNE1*	*NTRK1*	*CDKN2A*	*EGFR*
CDKN2A	*CTNNB1*	*DDR2*	*EGFR*	*CDK4*	*RET*	*GATA3*	*KIT*
ERBB2	*ESR1*	*EZH2*	*FBXW7*	*CDK6*	*ROS1*	*MET*	*MLH1*
FGFR1	*FGFR2*	*FGFR3*	*GATA3*	*EGFR*		*MTOR*	*NF1*
GNA11	*GNAQ*	*GNAS*	*HNF1A*	*ERBB2*		*PDGFRA*	*PTEN*
HRAS	*IDH1*	*IDH2*	*JAK2*	*FGFR1*		*RB1*	*SMAD4*
JAK3	*KIT*	*KRAS*	*MAP2K1*	*FGFR2*		*STK11*	*TP53*
MAP2K2	*MAPK1*	*MAPK3*	*MET*	*KIT*		*TSC1*	*VHL*
MLH1	*MPL*	*MTOR*	*MYC*	*KRAS*			
NF1	*NFE2L2*	*NOTCH1*	*NPM1*	*MET*			
NRAS	*NTRK1*	*NTRK3*	*PDGFRA*	*MYC*			
PIK3CA	*PTEN*	*PTPN11*	*RAF1*	*PDGFRA*			
RB1	*RET*	*RHEB*	*RHOA*	*PIK3CA*			
RIT1	*ROS1*	*SMAD4*	*SMO*	*RAF1*			
STK11	*TERT*	*TP53*	*TSC1*				
VHL							

TERT includes alterations in the promoter region. *MET* includes exon 14 skipping

Panel 1b. 70 gene panel of ctDNA (N=19 blood samples).

**POINT MUTATIONS**				**AMPLIFICATIONS**	**FUSIONS**	**INDELS**
AKT1	*ALK**	***APC***	***AR***	*AR*	*ALK*	*EGFR* exon 19 deletions
ARAF	***ARID1A***	*ATM*	***BRAF***	*BRAF*	*FGFR2*	*EGFR* exon 20 insertions
BRCA1	***BRCA2***	***CCND1***	***CCND2***	*CCND1*	*FGFR3*	*ERBB2* exon 19 deletions
CCNE1	*CDH1*	***CDK4***	***CDK6***	*CCND2*	*NTRK1*	*ERBB2* exon 20 insertions
CDKN2A	***CDKN2B***	*CTNNB1*	***EGFR***	*CCNE1*	*RET*	
ERBB2	*ESR1*	*EZH2*	*FBXW7*	*CDK4*	*ROS1*	
FGFR1	***FGFR2****	*FGFR3**	*GATA3*	*CDK6*		
GNA11	*GNAQ*	*GNAS*	*HNF1A*	*EGFR*		
HRAS	*IDH1*	*IDH2*	*JAK2*	*ERBB2*		
JAK3	***KIT***	***KRAS***	*MAP2K1*	*FGFR1*		
MAP2K2	***MET***	*MLH1*	*MPL*	*FGFR2*		
MYC	***NF1***	*NFE2L2*	*NOTCH1*	*KIT*		
NPM1	***NRAS***	*NTRK1**	***PDGFRA***	*KRAS*		
PIK3CA	***PTEN***	*PTPN11*	***RAF1***	*MET*		
RB1	*RET**	*RHEB*	*RHOA*	*MYC*		
RIT1	*ROS1**	*SMAD4*	*SMO*	*PDGFRA*		
SRC	*STK11*	*TERT*	***TP53***	*PIK3CA*		
TSC1	*VHL*			*RAF1*		

Complete exon and partial intron coverage for genes in ***bold***. *Genes with asterisk include rearrangements. *MET* includes exon 14 skipping.

Panel 1c. 68 gene panel of ctDNA (N=11 blood samples).

POINT MUTATIONS				AMPLIFICATIONS	FUSIONS	INDELS
AKT1	*ALK*	***APC***	***AR***	*AR*	*ALK*	*EGFR* exon 19 deletions
AFAR	***ARID1A***	*ATM*	***BRAF***	*BRAF*	*NTRK1*	*EGFR* exon 20 insertions
BRCA1	***BRCA2***	***CCDN1***	***CCDN2***	*CCNE1*	*RET*	
CCNE1	*CDH1*	***CDK4***	***CDK6***	*CDK4*	*ROS1*	
CDKN2A	***CDKN2B***	*CTNNB1*	***EGFR***	*CDK6*		
ERBB2	*ESR1*	*EZH2*	*FBXW7*	*EGFR*		
FGFR1	***FGFR2***	*FGFR3*	*GATA3*	*ERBB2*		
GNA11	*GNAQ*	*GNAS*	*HNF1A*	*FGFR1*		
HRAS	*IDH1*	*IDH2*	*JAK2*	*FGFR2*		
JAK3	***KIT***	***KRAS***	*MAP2K1*	*KIT*		
MAP2K2	***MET***	*MLH1*	*MPL*	*KRAS*		
MYC	***NF1***	*NFE2L2*	*NOTCH1*	*MET*		
NPM1	***NRAS***	*NTRK1*	***PDGFRA***	*MYC*		
PIK3CA	***PTEN***	*PTPN11*	***RAF1***	*PDGFRA*		
RET	*RHEB*	*RHOA*	*RIT1*	*PIK3CA*		
ROS1	*SMAD4*	*SMO*	*SRC*	*RAF1*		
STK11	*TERT*	***TP53***	*VHL*			

Complete exon coverage for genes in ***bold***.

Supplementary Table 2. Next-generation sequencing gene panels for tissue DNA (*Foundation Medicine, Inc.*) (N=90).
Panel 2a. 324 gene panel of tissue DNA (N=5 tissue samples).

**SUBSTITUTIONS, INSERTION/DELETIONS, AND COPY NUMBER ALTERATIONS**								
ABL1	*ACVR1B*	*AKT1*	*AKT2*	*AKT3*	*ALK*	*ALOX12B*	*AMER1*	*APC*
AR	*ARAF*	*ARFRP1*	*ARID1A*	*ASXL1*	*ATM*	*ATR*	*ATRX*	*AURKA*
AURKB	*AXIN1*	*AXL*	*BAP1*	*BARD1*	*BCL2*	*BCL2L1*	*BCL2L2*	*BCL6*
BCOR	*BCORL1*	*BRAF*	*BRCA1*	*BRCA2*	*BRD4*	*BRIP1*	*BTG1*	*BTG2*
BTK	*C11orf30*	*C17orf39*	*CALR*	*CARD11*	*CASP8*	*CBFB*	*CBL*	*CCND1*
CCND2	*CCND3*	*CCNE1*	*CD22*	*CD274*	*CD70*	*CD79A*	*CD79B*	*CDC73*
CDH1	*CDK12*	*CDK4*	*CDK6*	*CDK8*	*CDKN1A*	*CDKN1B*	*CDKN2A*	*CDKN2B*
CDKN2C	*CEBPA*	*CHEK1*	*CHEK2*	*CIC*	*CREBBP*	*CRKL*	*CSF1R*	*CSF3R*
CTCF	*CTNNA1*	*CTNNB1*	*CUL3*	*CUL4A*	*CXCR4*	*CYP17A1*	*DAXX*	*DDR1*
DDR2	*DIS3*	*DNMT3A*	*DOT1L*	*EED*	*EGFR*	*EP300*	*EPHA3*	*EPHB1*
EPHB4	*ERBB2*	*ERBB3*	*ERBB4*	*ERCC4*	*ERG*	*ERRFI1*	*ESR1*	*EZH2*
FAM46C	*FANCA*	*FANCC*	*FANCG*	*FANCL*	*FAS*	*FBXW7*	*FGF10*	*FGF12*
FGF14	*FGF19*	*FGF23*	*FGF3*	*FGF4*	*FGF6*	*FGFR1*	*FGFR2*	*FGFR3*
FGFR4	*FH*	*FLCN*	*FLT1*	*FLT3*	*FOXL2*	*FUBP1*	*GABRA6*	*GATA3*
GATA4	*GATA6*	*GNA11*	*GNA13*	*GNAQ*	*GNAS*	*GRM3*	*GSK3B*	*H3F3A*
HDAC1	*HGF*	*HNF1A*	*HRAS*	*HSD3B1*	*ID3*	*IDH1*	*IDH2*	*IGF1R*
IKBKE	*IKZF1*	*INPP4B*	*IRF2*	*IRF4*	*IRS2*	*JAK1*	*JAK2*	*JAK3*
JUN	*KDM5A*	*KDM5C*	*KDM6A*	*KDR*	*KEAP1*	*KEL*	*KIT*	*KLHL6*
KMT2A	*KMT2D*	*KRAS*	*LTK*	*LYN*	*MAF*	*MAP2K1*	*MAP2K2*	*MAP2K4*
MAP3K1	*MAP3K13*	*MAPK1*	*MCL1*	*MDM2*	*MDM4*	*MED12*	*MEF2B*	*MEN1*
MERTK	*MET*	*MITF*	*MKNK1*	*MLH1*	*MPL*	*MRE11A*	*MSH2*	*MSH3*
MSH6	*MST1R*	*MTAP*	*MTOR*	*MUTYH*	*MYC*	*MYCL*	*MYCN*	*MYD88*
NBN	*NF1*	*NF2*	*NFE2L2*	*NFKBIA*	*NKX2-1*	*NOTCH1*	*NOTCH2*	*NOTCH3*
NPM1	*NRAS*	*NSD3*	*NT5C2*	*NTRK1*	*NTRK2*	*NTRK3*	*P2RY8*	*PALB2*
PARK2	*PARP1*	*PARP2*	*PARP3*	*PAX5*	*PBRM1*	*PDCD1*	*PDCD1LG2*	*PDGFRA*
PDGFRB	*PDK1*	*PIK3C2B*	*PIK3C2G*	*PIK3CA*	*PIK3CB*	*PIK3R1*	*PIM1*	*PMS2*
POLD1	*POLE*	*PPARG*	*PPP2R1A*	*PPP2R2A*	*PRDM1*	*PRKAR1A*	*PRKCI*	*PTCH1*
PTEN	*PTPN11*	*PTPRO*	*QKI*	*RAC1*	*RAD21*	*RAD51*	*RAD51B*	*RAD51C*
RAD51D	*RAD52*	*RAD54L*	*RAF1*	*RARA*	*RB1*	*RBM10*	*REL*	*RET*
RICTOR	*RNF43*	*ROS1*	*RPTOR*	*SDHA*	*SDHB*	*SDHC*	*SDHD*	*SETD2*
SF3B1	*SGK1*	*SMAD2*	*SMAD4*	*SMARCA4*	*SMARCB1*	*SMO*	*SNCAIP*	*SOCS1*
SOX2	*SOX9*	*SPEN*	*SPOP*	*SRC*	*STAG2*	*STAT3*	*STK11*	*SUFU*
SYK	*TBX3*	*TEK*	*TET2*	*TGFBR2*	*TIPARP*	*TNFAIP3*	*TNFRSF14*	*TP53*
TSC1	*TSC2*	*TYRO3*	*U2AF1*	*VEGFA*	*VHL*	*WHSC1*	*WT1*	*XPO1*
XRCC2	*ZNF217*	*ZNF703*						
**REARRANGEMENTS**								
ALK	*BCL2*	*BCR*	*BRAF*	*BRCA1*	*BRCA2*	*CD74*	*EGFR*	*ETV4*
ETV5	*ETV6*	*EWSR1*	*EZR*	*FGFR1*	*FGFR2*	*FGFR3*	*KIT*	*KMT2A*
MSH2	*MYB*	*MYC*	*NOTCH2*	*NTRK1*	*NTRK2*	*NUTM1*	*PDGFRA*	*RAF1*
RARA	*RET*	*ROS1*	*RSPO2*	*SDC4*	*SLC34A2*	*TERC*	*TERT**	*TMPRSS2*

*promoter region

Panel 2b. 315 gene panel of tissue DNA (N=72 tissue samples).

**SUBSTITUTIONS, INSERTION/DELETIONS, AND COPY NUMBER ALTERATIONS**								
ABL1	*ABL2*	*ACVR1B*	*AKT1*	*AKT2*	*AKT3*	*ALK*	*AMER1*	*APC*
AR	*ARAF*	*ARFRP1*	*ARID1A*	*ARID1B*	*ARID2*	*ASXL1*	*ATM*	*ATR*
ATRX	*AURKA*	*AURKB*	*AXIN1*	*AXL*	*BAP1*	*BARD1*	*BCL2*	*BCL2L1*
BCL2L2	*BCL6*	*BCOR*	*BCORL1*	*BLM*	*BRAF*	*BRCA1*	*BRCA2*	*BRD4*
BRIP1	*BTG1*	*BTK*	*C11orf30*	*CARD11*	*CBFB*	*CBL*	*CCND1*	*CCND2*
CCND3	*CCNE1*	*CD274*	*CD79A*	*CD79B*	*CDC73*	*CDH1*	*CDK12*	*CDK4*
CDK6	*CDK8*	*CDKN1A*	*CDKN1B*	*CDKN2A*	*CDKN2B*	*CDKN2C*	*CEBPA*	*CHD2*
CHD4	*CHEK1*	*CHEK2*	*CIC*	*CREBBP*	*CRKL*	*CRLF2*	*CSF1R*	*CTCF*
CTNNA1	*CTNNB1*	*CUL3*	*CYLD*	*DAXX*	*DDR2*	*DICER1*	*DNMT3A*	*DOT1L*
EGFR	*EP300*	*EPHA3*	*EPHA5*	*EPHA7*	*EPHB1*	*ERBB2*	*ERBB3*	*ERBB4*
ERG	*ERRFI1*	*ESR1*	*EZH2*	*FAM46C*	*FANCA*	*FANCC*	*FANCD2*	*FANCE*
FANCF	*FANCG*	*FANCL*	*FAS*	*FAT1*	*FBXW7*	*FGF10*	*FGF14*	*FGF19*
FGF23	*FGF3*	*FGF4*	*FGF6*	*FGFR1*	*FGFR2*	*FGFR3*	*FGFR4*	*FH*
FLCN	*FLT1*	*FLT3*	*FLT4*	*FOXL2*	*FOXP1*	*FRS2*	*FUBP1*	*GABRA6*
GATA1	*GATA2*	*GATA3*	*GATA4*	*GATA6*	*GID4*	*GLI1*	*GNA11*	*GNA13*
GNAQ	*GNAS*	*GPR124*	*GRIN2A*	*GRM3*	*GSK3B*	*H3F3A*	*HGF*	*HNF1A*
HRAS	*HSD3B1*	*HSP90AA1*	*IDH1*	*IDH2*	*IGF1R*	*IGF2*	*IKBKE*	*IKZF1*
IL7R	*INHBA*	*INPP4B*	*IRF2*	*IRF4*	*IRS2*	*JAK1*	*JAK2*	*JAK3*
JUN	*KAT6A*	*KDM5A*	*KDM5C*	*KDM6A*	*KDR*	*KEAP1*	*KEL*	*KIT*
KLHL6	*KMT2A*	*KMT2C*	*KMT2D*	*KRAS*	*LMO1*	*LRP1B*	*LYN*	*LZTR1*
MAGI2	*MAP2K1*	*MAP2K2*	*MAP2K4*	*MAP3K1*	*MCL1*	*MDM2*	*MDM4*	*MED12*
MEF2B	*MEN1*	*MET*	*MITF*	*MLH1*	*MPL*	*MRE11A*	*MSH2*	*MSH6*
MTOR	*MUTYH*	*MYC*	*MYCL*	*MYCN*	*MYD88*	*NF1*	*NF2*	*NFE2L2*
NFKBIA	*NKX2-1*	*NOTCH1*	*NOTCH2*	*NOTCH3*	*NPM1*	*NRAS*	*NSD1*	*NTRK1*
NTRK2	*NTRK3*	*NUP93*	*PAK3*	*PALB2*	*PARK2*	*PAX5*	*PBRM1*	*PDCD1LG2*
PDGFRA	*PDGFRB*	*PDK1*	*PIK3C2B*	*PIK3CA*	*PIK3CB*	*PIK3CG*	*PIK3R1*	*PIK3R2*
PLCG2	*PMS2*	*POLD1*	*POLE*	*PPP2R1A*	*PRDM1*	*PREX2*	*PRKAR1A*	*PRKCI*
PRKDC	*PRSS8*	*PTCH1*	*PTEN*	*PTPN11*	*QKI*	*RAC1*	*RAD50*	*RAD51*
RAF1	*RANBP2*	*RARA*	*RB1*	*RBM10*	*RET*	*RICTOR*	*RNF43*	*ROS1*
RPTOR	*RUNX1*	*RUNX1T1*	*SDHA*	*SDHB*	*SDHC*	*SDHD*	*SETD2*	*SF3B1*
SLIT2	*SMAD2*	*SMAD3*	*SMAD4*	*SMARCA4*	*SMARCB1*	*SMO*	*SNCAIP*	*SOCS1*
SOX10	*SOX2*	*SOX9*	*SPEN*	*SPOP*	*SPTA1*	*SRC*	*STAG2*	*STAT3*
STAT4	*STK11*	*SUFU*	*SYK*	*TAF1*	*TBX3*	*TERC*	*TERT**	*TET2*
TGFBR2	*TNFAIP3*	*TNFRSF14*	*TOP1*	*TOP2A*	*TP53*	*TSC1*	*TSC2*	*TSHR*
U2AF1	*VEGFA*	*VHL*	*WISP3*	*WT1*	*XPO1*	*ZBTB2*	*ZNF217*	*ZNF703*
**REARRANGEMENTS**								
ALK	*BCL2*	*BCR*	*BRAF*	*BRCA1*	*BRCA2*	*BRD4*	*EGFR*	*ETV1*
ETV4	*ETV5*	*ETV6*	*FGFR1*	*FGFR2*	*FGFR3*	*KIT*	*MSH2*	*MYB*
MYC	*NOTCH2*	*NTRK1*	*NTRK2*	*PDGFRA*	*RAF1*	*RARA*	*RET*	*ROS1*
TMPRSS2								

*promoter region

Panel 2c. 236 gene panel of tissue DNA (N=13 tissue samples).

**SUBSTITUTIONS, INSERTION/DELETIONS, AND COPY NUMBER ALTERATIONS**								
ABL1	*AKT1*	*AKT2*	*AKT3*	*ALK*	*APC*	*AR*	*ARAF*	*ARFRP1*
ARID1A	*ARID2*	*ASXL1*	*ATM*	*ATR*	*ATRX*	*AURKA*	*AURKB*	*AXL*
BAP1	*BARD1*	*BCL2*	*BCL2L2*	*BCL6*	*BCOR*	*BCORL1*	*BLM*	*BRAF*
BRCA1	*BRCA2*	*BRIP1*	*BTK*	*C11orf30*	*C17orf39*	*CARD11*	*CBFB*	*CBL*
CCND1	*CCND2*	*CCND3*	*CCNE1*	*CD79A*	*CD79B*	*CDC73*	*CDH1*	*CDK12*
CDK4	*CDK6*	*CDK8*	*CDKN1B*	*CDKN2A*	*CDKN2B*	*CDKN2C*	*CEBPA*	*CHEK1*
CHEK2	*CIC*	*CREBBP*	*CRKL*	*CRLF2*	*CSF1R*	*CTCF*	*CTNNA1*	*CTNNB1*
DAXX	*DDR2*	*DNMT3A*	*DOT1L*	*EGFR*	*EP300*	*EPHA3*	*EPHA5*	*EPHB1*
ERBB2	*ERBB3*	*ERBB4*	*ERG*	*ESR1*	*EZH2*	*FAM123B*	*FAM46C*	*FANCA*
FANCC	*FANCD2*	*FANCE*	*FANCF*	*FANCG*	*FANCL*	*FBXW7*	*FGF10*	*FGF14*
FGF19	*FGF23*	*FGF3*	*FGF4*	*FGF6*	*FGFR1*	*FGFR2*	*FGFR3*	*FGFR4*
FLT1	*FLT3*	*FLT4*	*FOXL2*	*GATA1*	*GATA2*	*GATA3*	*GNA11*	*GNA13*
GNAQ	*GNAS*	*GPR124*	*GRIN2A*	*GSK3B*	*HGF*	*HRAS*	*IDH1*	*IDH2*
IGF1R	*IKBKE*	*IKZF1*	*IL7R*	*INHBA*	*IRF4*	*IRS2*	*JAK1*	*JAK2*
JAK3	*JUN*	*KAT6A*	*KDM5A*	*KDM5C*	*KDM6A*	*KDR*	*KEAP1*	*KIT*
KLHL6	*KRAS*	*LRP1B*	*MAP2K1*	*MAP2K2*	*MAP2K4*	*MAP3K1*	*MCL1*	*MDM2*
MDM4	*MED12*	*MEF2B*	*MEN1*	*MET*	*MITF*	*MLH1*	*MLL*	*MLL2*
MPL	*MRE11A*	*MSH2*	*MSH6*	*MTOR*	*MUTYH*	*MYC*	*MYCL1*	*MYCN*
MYD88	*NF1*	*NF2*	*NFE2L2*	*NFKBIA*	*NKX2-1*	*NOTCH1*	*NOTCH2*	*NPM1*
NRAS	*NTRK1*	*NTRK2*	*NTRK3*	*NUP93*	*PAK3*	*PALB2*	*PAX5*	*PBRM1*
PDGFRA	*PDGFRB*	*PDK1*	*PIK3CA*	*PIK3CG*	*PIK3R1*	*PIK3R2*	*PPP2R1A*	*PRDM1*
PRKAR1A	*PRKDC*	*PTCH1*	*PTEN*	*PTPN11*	*RAD50*	*RAD51*	*RAF1*	*RARA*
RB1	*RET*	*RICTOR*	*RNF43*	*RPTOR*	*RUNX1*	*SETD2*	*SF3B1*	*SMAD2*
SMAD4	*SMARCA4*	*SMARCB1*	*SMO*	*SOCS1*	*SOX10*	*SOX2*	*SPEN*	*SPOP*
SRC	*STAG2*	*STAT4*	*STK11*	*SUFU*	*TET2*	*TGFBR2*	*TNFAIP3*	*TNFRSF14*
TOP1	*TP53*	*TSC1*	*TSC2*	*TSHR*	*VHL*	*WISP3*	*WT1*	*XPO1*
ZNF217	*ZNF703*							
**REARRANGEMENTS**								
ALK	*BCL2*	*BCR*	*BRAF*	*EGFR*	*ETV1*	*ETV4*	*ETV5*	*ETV6*
EWSR1	*MLL*	*MYC*	*NTRK1*	*PDGFRA*	*RAF1*	*RARA*	*RET*	*ROS1*
TMPRSS2								

Supplementary Table 3. List of potential targeted therapies with FDA-approved agents (on- or off-label).

Altered Genes	Potential targeted therapies with FDA approved agents	References
AKT1/2/3	*AKT* is potentially targetable with mTOR inhibitors such as everolimus.	1-4
APC	*APC* alteration is potentially targetable with COX-2 inhibitors such as celecoxib.	5-7
AR	*AR* alteration is potentially targetable with AR inhibitor enzalutamide and anti-androgens (e.g., abiraterone, bicalutamide).	8, 9
ARAF	*ARAF* alteration is potentially targetable with sorafenib.	10
ATM	*ATM* alteration is potentially targetable with PARP inhibitor (e.g., olaparib, niraparib, rucaparib).	11-13
AXL	*AXL* alteration is potentially targetable with multi-kinase inhibitor cabozantinib.	14
BAP1	*BAP1* (*BRCA1-associated protein-1*) alteration is potentially targetable with PARP inhibitor such as olaparib.	15, 16
BCL2	*BCL2* is potentially targetable with Bcl-2 inhibitors such as venetoclax.	17
BRAF	*BRAF* alteration can be targeted with BRAF inhibitors (e.g. dabrafenib, vemurafenib) and MEK inhibitors (e.g. trametinib, cobimetinib).	18, 19
BRCA1/2	*BRCA* alteration is targetable with platinum-based chemotherapy and PARP inhibitor (e.g., olaparib, niraparib, rucaparib).	20, 21
BRIP1	*BRIP1* encodes BRCA1-interacting protein-1 which is potentially targetable with PARP inhibitor such as olaparib.	16
CCND1/2/3	Although data are conflicting, *CCND1/2/3* aberrations are theoretically targetable with CDK4/6 inhibitors such as palbociclib.	22-24
CCNE1	*CCNE1* aberration is potentially targetable with proteasome inhibitors such as bortezomib.	25
CDK4/6	*CDK6* and its functional homolog, *CDK4* mutations are theoretically targetable with CDK4/6 inhibitors (e.g., palbociclib, abemaciclib, ribociclib).	26, 27
CDKN2A/B	Although there are conflicting report, *CDKN1/2* alterations are theoretically targetable with CDK4/6 inhibitors such as palbociclib.	22, 23
CHEK2	*CHEK2* is DNA-repair gene. Alteration in *CHEK2* is potentially actionable with PARP inhibitor such as olaparib.	12
CTNNB1	*CTNNB1* (beta-catenin) is part of Wnt signaling. Sorafenib was shown to have anti-cancer effect by modulating Wnt/beta-catenin signaling (preclinical data).	28
DNMT3A	*DNMT3A* alteration is potentially targetable with decitabine.	29
EGFR	*EGFR* alteration is targetable with EGFR inhibitors including afatinib and erlotinib.	30-32
ERBB2	*ERBB2* alteration is targetable with Her-targeted drugs (e.g., afatinib, lapatinib, neratinib, pertuzumab, trastuzumab).	33-36
ERBB3/4	*ERBB3* is potentially targetable with afatinib.	37
ESR1	ESR1 encodes estrogen receptor and is targetable with aromatase inhibitors and estrogen receptor antagonists.	38
FANCA/FANCL	*FANC* genes are associated with DNA repair. Alterations in *FANC* genes are potentially targetable with PARP inhibitors such as olaparib.	12
FBXW7	Although there are conflicting data, FBXW7 aberration stabilizes the mTOR signaling which is potentially targetable with mTOR inhibitors (e.g., everolimus, temsirolimus).	39-41

FGF/FGFR	*FGF* and *FGFR* alterations are targetable with multi-kinase inhibitors such as lenvatinib.	42, 43
FLT3/4	*FLT3* alteration is targetable with midostaurin, ponatinib, sorafenib, and sunitinib. *FLT4* is potentially targetable with VEGFR3 inhibitors such as axitinib and sorafenib.	44-49
FRS2	*FRS2* alteration is potentially targetable with multi-kinase inhibitor such as lenvatinib.	50
GNAS	*GNAS* alteration is potentially targetable with MEK inhibitor such as trametinib or cobimetinib.	51-54
HGF	*HGF* is potentially targetable with multi-kinase inhibitors that includes MET inhibition such as cabozantinib.	55
IDH1	*IDH1* alteration is targetable with IDH1 inhibitors (e.g., ivosidenib).	56, 57
JAK2	*JAK2* alterations are potentially targetable with ruxolitinib.	58
KRAS	*KRAS* alteration is potentially targetable with MEK inhibitor such as trametinib or cobimetinib.	51, 59-64
MET	*MET* alteration is targetable with multi-kinase inhibitors such as cabozantinib and crizotinib.	65
MLH1	Alterations in *MLH1, a* mismatch-repair gene, result in high tumor mutational burden targetable by immunotherapy with checkpoint inhibitors (nivolumab, pembrolizumab).	66, 67
NF1	*NF1* alteration is associated with activation of RAS and downstream pathways. Thus, it may be targetable with MEK inhibitors (e.g., trametinib, cobimetinib).	53, 54, 68, 69
NF2	*NF2* is negative regulator of mTOR, thus it is potentially targetable with mTOR inhibitor such as everolimus and temsirolimus.	70
NRAS	*KRAS* alteration is potentially targetable with MEK inhibitor such as trametinib or cobimetinib.	51, 64
PDCD1LG2	*PDCD1LG2 (PD-L2)* is targetable with checkpoint inhibitors such as nivolumab and pembrolizumab.	71, 72
PDGFRA	*PDGFR* alteration is targetable with multi-kinase inhibitors such as dasatinib, imatinib or sunitinib.	73
PIK3CA	*PIK3CA* alteration is targetable with mTOR inhibitors such as everolimus and temsirolimus.	1, 2
PIK3R1	*PIK3R1* alteration is potentially targetable with an mTOR inhibitor such as everolimus and temsirolimus.	74
PTCH1	*PTCH1* alteration is targetable with SMO inhibitor vismodegib.	75
PTEN	*PTEN* alteration is targetable with mTOR inhibitors such as everolimus and temsirolimus.	1, 2, 76
RAD50	*RAD50* is a gene associated with DNA double-strand break repair. Alteration in *RAD50* is potentially actionable with PARP inhibitor such as olaparib.	77-79
SRC	*SRC* alteration may be targetable with dasatinib.	80
STK11	*STK11* alteration contributes to mTORC1 activation and thus it is potentially targetable with mTORC1 inhibitor such as everolimus or temsirolimus.	81, 82
TP53	Retrospective data suggest patients with *TP53* mutation had longer progression-free survival with bevacizumab containing regimen when compared to non-bevacizumab containing regimen (median 11.0 vs. 4.0 months, $p<0.0001$). *TP53* alteration status was also predictive of longer progression-free survival among sarcoma patients treated with pazopanib (multi-kinase inhibitor including VEGF) (hazard ratio: 0.38, $p = 0.036$). Interestingly, multiple regression analysis of transcriptomic data revealed *TP53* mutations are associated with higher *VEGF-A* expression ($p=0.0006$) suggesting the *TP53* as a marker to predict bevacizumab response.	83-86
VEGFA	*VEGFA* is targetable with anti-VEGF therapy including bevacizumab.	87

Supplementary Table 4. Detailed information of patients with biliary tract cancers who were treated with matched targeted therapies based on molecular profiling (N=34).

ID	Age/ Sex	Tumor type	Tissue DNA sequencing prior to therapy	ctDNA sequencing prior to therapy	Treatment regimen	Line of regimen	PFS (months)*	Best response
**4**	78/M	IHCC	*IDH1* R132C, *FBXW7* R46fs*1, *BAP1* Y401fs*1	*Not reported	gemcitabine, cisplatin	2nd line (1st-line: 5FU)	5.8	SD
**6**	74/F	GBCA	*STK11* splice site 921-1G>C, *CDKN2A* loss, *CHEK2* L467*, *TGFBR2* D524N	*Not reported	gemcitabine, cisplatin	1st line	3.8	PD
**12**	60/M	EHCC	*BRCA2* R2842C, *KRAS* G12V, *RBM10* F178fs*88	No characterized alteration	gemcitabine, cisplatin	1st line	5.9	PD
**13**	68/F	GBCA	*BRCA2* N319fs*8, *PIK3R1* splice site 1300-11_1308del20, *PTEN* V45fs*7, *CDKN2A* splice site 151-1_203del54, *ARID1A* E2250*, *MUTYH* G382D	*PIK3CA* amplification	carboplatin, everolimus	1st line	4.8	SD
**23**	48/F	IHCC	*KRAS* Q61H, *PIK3CA* H1047Y	*ESR1* C530S, *KRAS* Q61H, *PIK3CA* E545K	trametinib, letrozole	5th line (1st-line: gemcitabine, cisplatin; 2nd: 5FU, irinotecan, oxaliplatin; 3rd: ipilimumab, pembrolizumab; 4th: lenvatinib, pembrolizumab)	1.1+	Not restaged yet
**27**	59/F	IHCC	*FLT4* R1223H, *IDH1* R132L, *BAP1* W52*, *CDKN2A/B* loss, *SETD2* splice site 4918-2A>C<br><br>PD-L1 on IHC (low positive)	*Not reported	pembrolizumab	1st line	2.1	PD
**29**	46/F	IHCC	*FGFR2-BICC2* fusion, *POLE* R446Q	*PIK3CA* amplification	everolimus, lenvatinib	2nd line (1st-line: gemcitabine, cisplatin)	3.9	SD
**31**	77/M	EHCC	*MDM2* amplification, *CDKN2A* p16INK4a R80* and p14ARF P94L,	No characterized alteration	lenvatinib, palbociclib	1st line	11.5	PR

			CEBPA G103_G104del, *FRS2* amplification					
**32**	42/F	IHCC	*BRIP1* R581*, *MAP2K4* loss, *TNFRSF14* E44fs*20, *TP53* G199E	*Not reported	capecitabine, oxaliplatin	1st line	5.6	SD
**33**	33/F	IHCC	*FGFR2-BICC1* fusion	*FGFR2-BICC1* fusion, *KRAS* amplification	BGJ398**	2nd line (1st-line: gemcitabine, cisplatin)	16.8	SD
**34**	83/M	EHCC	*BRCA2* S1630*, *ERBB2* amplification, *KRAS* G12D, *VEGFA* amplification, *INPP4B* truncation intron 23, *TP53* R342*, *AXIN1* W85	*TP53* R342*	gemcitabine, cisplatin	1st line	6.5	SD
**35**	74/F	GBCA	*CDK4* amplification, *ATM* F2558fs*5, *ATM* Q445*, *MDM2* amplification, *APC* G1735fs*34, *ARID2* S1057*, *FRS2* amplification, *KEAP1* loss	*Not reported	gemcitabine, cisplatin	1st line	5.6	SD
**37**	82/M	IHCC	*KRAS* G12D, *MLH1* splice site 1989+1G>T, *TP53* R248Q<br><br>PD-L1 on IHC (low positive)	*CCND2* C46*, *TP53* R248Q, *KRAS* G12D, *MLH1* R389W	nivolumab, trametinib	1st line	15.8	PR
**38**	58/F	IHCC	*KRAS* G12A, *IDH1* R132C, *ARID1A* S402*, *BAP1* loss exon 3, *FOXP1* loss, *TET2* C1271*	*Not reported	bevacizumab, ivosidenib, trametinib	1st line	3.1	PD
**39**	53/M	C-HCC	*TP53* V157F, *JUN* amplification	No characterized alteration	sorafenib	2nd line (1st-line: 5FU)	3.1	PD
**41**	63/F	IHCC	*STK11* loss, *IDH1* R132L	*Not reported	everolimus	1st line	2.5	PD
**44**	61/F	GBCA	*AKT2* amplification, *CCND1* amplification, *CDKN2A/B* loss, *SMAD4* loss, *TP53* R175H	No characterized alteration	everolimus, palbociclib	1st line	3.2	PD
**47**	66/F	IHCC	*PTCH1* V1131M, *ARID1A* R1026fs*13, BAP1 D672G, CDKN2A/B loss	*FGFR1* amplification	gemcitabine, cisplatin	1st line	2.6	PD

**51**	60/F	EHCC	*FBXW7* E117del, *STK11* E33*, *STK11* splice site 723_734+13del25, *ARID1A* A147fs*253, *CDKN2A* splice site 151-14_154del18, *RBM10* M1?	*STK11* c.723_734+13del	everolimus, lenvatinib, palbociclib	4th line (1st-line: gemcitabine, cisplatin; 2nd: capecitabine; 3rd: everolimus, metformin)	2.3	PD
**52**	60/M	IHCC	*KRAS* G12D, *TP53* C135Y	*KRAS* G12D, *TERT* Promoter SNV, *TP53* R337C	afatinib, anakinra, trametinib	2nd line (1st-line: gemcitabine, oxaliplatin)	1.6	PD
**54**	61/F	GBCA	*CCNE1* amplification, *MLL3* C310S, *MLL3* R4225*<br><br>PD-L1 on IHC (low positive)	*TP53* G266E, *TP53* G244D	nivolumab	2nd line (1st-line: gemcitabine, cisplatin)	11.7	PR
**56**	78/M	EHCC	*CDKN2A/B* loss, *KDM6A* loss exons 3-26, *KDM6A* Q1304*, *TET2* L1212*, *TET2* T938fs*33, *TP53* R248L<br><br>TMB (9 Muts/Mb, intermediate)	No characterized alteration	palbociclib, pembrolizumab	2nd line (1st-line: capecitabine)	2.9	SD
**59**	63/M	IHCC	*KRAS* G12D, *CDKN2A* p16INK4a deletion exons 1-2 and p14ARF deletion exon 2, *GNAS* R201H, *RBM10* E393*<br><br>PD-L1 on IHC (unknown)	*GNAS* R201H, *ARID1A* K1125fs, *KRAS* G12D	nivolumab, palbociclib, trametinib	2nd line (1st-line: gemcitabine, cisplatin)	3.9	SD
**64**	36/M	GBCA	*ERBB2* amplification, *CDK6* amplification, *MYC* amplification, *CDKN2A/B* loss, *FANCA* V372fs*42, *KMT2C* (MLL3) E1689fs*28, *TP53* N247I	*Not reported	pertuzumab, trastuzumab	1st line	18.3+	PR
**66**	62/F	IHCC	*FGFR2* duplication exons 7 -17, *PIK3CA* E542K, *BRAF* amplification, *EZH2* loss exons 4-20, *FANCL* M1V, *FAT1* S1310*	*KRAS* G12V, *KRAS* G12R, *TP53* R282W, *TP53* N239D, *PIK3CA* E542K, *PIK3CA* H1047R, *PIK3CA* E545K, *BRAF*	gemcitabine, cisplatin	1st line	10.2	PR

				amplification, *CCNE1* amplification				
**67**	56/F	IHCC	*ATM* Y2437fs*12, *EMSY* amplification, *MCL1* amplification	No characterized alteration	gemcitabine, cisplatin	1st line	4.3	PR
**71**	60/F	IHCC	*Not reported<br><br>PD-L1 IHC (negative)	*TP53* T263P, *ARID1A* Q372fs	bevacizumab, nivolumab	1st line	0.7	PD
**75**	69/F	GBCA	*Not reported	*ERBB2* amplification, *TP53* R282W	bevacizumab, lapatinib, trastuzumab	1st line	10.4+	PR
**86**	66/M	IHCC	*FBXW7* R543fs*8, *KRAS* G12D, *CDKN2A/B* loss, *RAD50* S143Y, *TERC* amplification, *TP53* P151T	*Not reported	gemcitabine, oxaliplatin	1st line	5.7+	PR
**92**	60/M	GBCA	ERBB2 L313V, APC R2237*, CDKN2A/B loss, MAP2K4 S184L	*APC* R2237*	afatinib, palbociclib, trastuzumab	1st line	2.5	PD
**99**	31/F	IHCC	*FGFR2-TMPO* fusion, *BAP1* S58_S70>D, *ARID1A* K327fs*68, *PBRM1* W992*, *SMARCA4* E523*	*Not reported	gemcitabine, oxaliplatin	2nd line (1st-line: carboplatin, paclitaxel, etoposide)	10.6	SD
**101**	65/M	C-HCC	*CCND1* amplification, *TP53* R273S, *FGF19* amplification, *FGF3* amplification, *FGF4* amplification, *TERT* promoter -124C>T	*Not reported	sorafenib	1st line	2.8	PD
**117**	43/F	IHCC	*Not reported<br><br>PD-L1 on IHC (low positive)	*CDK4* amplification, *MYC* amplification	pembrolizumab	1st line	2.9+	SD
**118**	78/F	IHCC	*ARID1A* F1823fs*3, *BAP1* loss exons 1-10, *CDKN2A/B* loss, *MTAP* loss, *PBRM1* loss exons 19-30	*Not reported	gemcitabine, oxaliplatin	1st line	3.7+	SD

* + sign in PFS indicates ongoing response.

** A FGFR1/2/3 inhibitor.

Abbreviations: C-HCC, mixed cholangio-hepatocellular carcinoma; EHCC, extrahepatic cholangiocarcinoma; GBCA, gallbladder adenocarcinoma; IHC, immunohistochemistry; IHCC, intrahepatic cholangiocarcinoma; PD, progressive disease; PD-L1, programmed death-ligand 1; PR, partial response; SD, stable disease; TMB, tumor mutational burden.

Supplementary Table 5. Basic characteristics of the matched versus unmatched patients [N=80].

Characteristics	***Treatment group*** *Matched therapy (N=34)*	*Unmatched therapy (N=46)*	*P-value*
**Age*, years**			
Median age (range)	60.9 (31.2-82.6)	64.0 (38.4-88.5)	0.58
≥63, n (%)	13 (38.2%)	24 (52.2%)	0.26
**Sex, n (%)**			
Male	13 (38.2%)	28 (60.9%)	0.07
Female	21 (61.8%)	18 (39.1%)	
**ECOG-PS ≥2, n (%)**	10 (29.4%)	11 (23.9%)	0.62
**Total bilirubin >3.6mg/dL****	3 (8.8%)	5 (10.9%)	>0.99
**Tumor site, n (%)**			
IHCC	19 (55.9%)	22 (47.8%)	0.51
EHCC	5 (14.7%)	12 (26.1%)	0.28
GBCA	8 (23.5%)	9 (19.6%)	0.78
C-HCC	2 (5.9%)	3 (6.5%)	>0.99
**Extent of disease at the time start, n (%)**			
Metastatic / locally advanced	30 / 4 (88.2% / 11.8%)	37 / 9 (80.4% / 19.6%)	0.54
Extent to extrahepatic	29 (85.3%)	34 (73.9%)	0.28
Lung metastasis	5 (14.7%)	12 (26.1%)	0.28
Peritoneal metastasis	15 (44.1%)	11 (23.9%)	0.09
**History of radical surgery, n (%)**	13 (38.2%)	17 (37.0%)	>0.99
**Line of the regimen, n (%)**			
Administered as 1st line	23 (67.6%)	39 (84.8%)	0.10
**Median time to initiation of the treatment, months (range)*****	2.9 (0.3-35.5)	1.5 (0.0-41.5)	**0.04**

* Age at diagnosis

** Total bilirubin at the time of treatment start. Dichotomized by (3 x institutional upper limit of normal [1.2 mg/dL]).

*** Time from advanced disease diagnosis to initiation of the treatment.

Abbreviations: C-HCC, cholangio-hepatocellular carcinoma; ctDNA, circulating-tumor DNA; ECOG-PS, Eastern Cooperative Oncology Group Performance Status; EHCC, extrahepatic cholangiocarcinoma; GBCA, gallbladder carcinoma; IHCC, intrahepatic cholangiocarcinoma; PFS, progression-free survival.

Supplementary Figure 1. Consort flow chart (N=121).

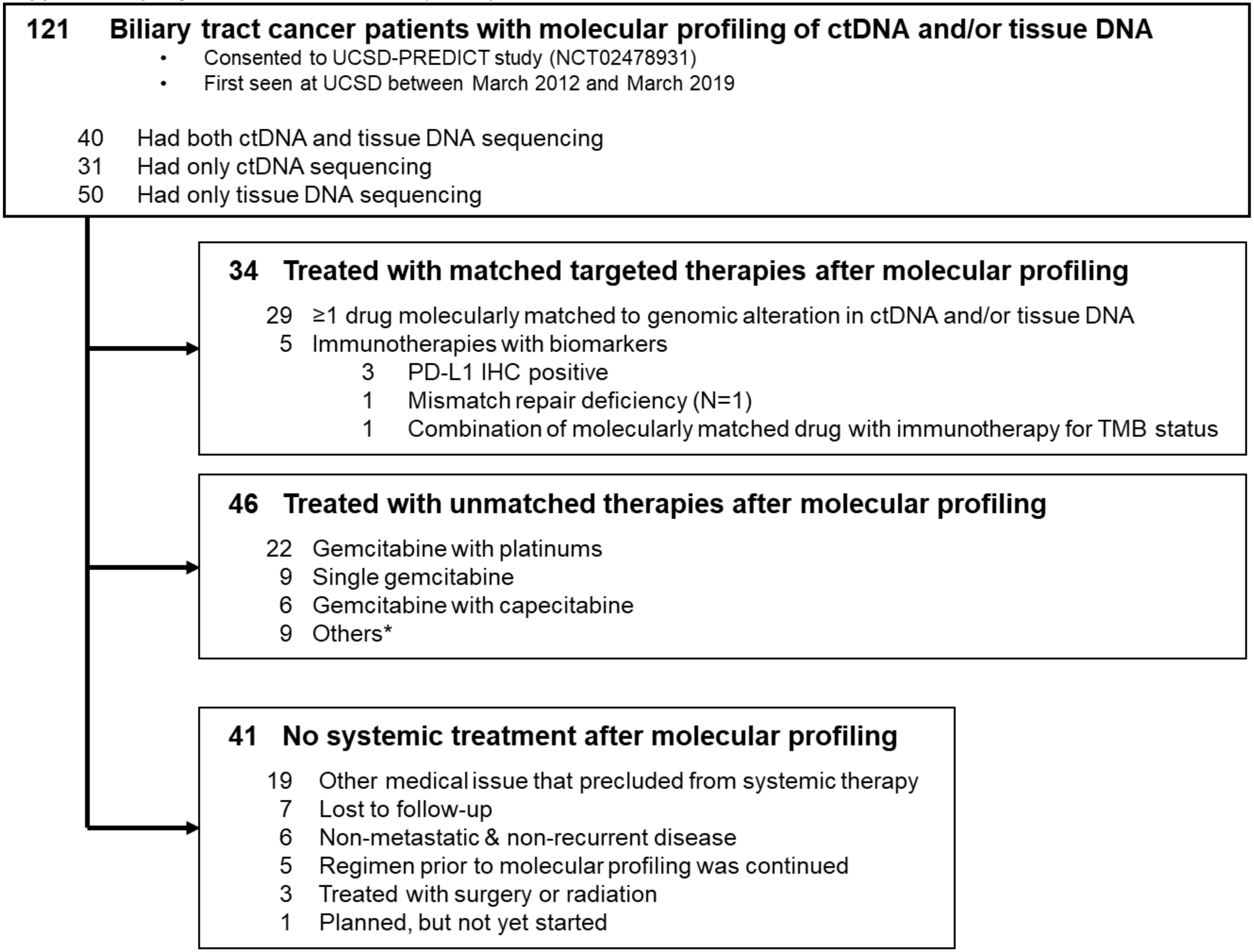

*Others: FOLFOX/CapeOX [N=4]; single capecitabine [N=1]; pembrolizumab [N=1]; sorafenib [N=1]; pertuzumab, bevacizumab, plus afatinib [N=1]; and cisplatin plus vinblastine [N=1].

Abbreviations: ctDNA, circulating tumor DNA, IHC, immunohistochemistry assay; PD-L1, programmed death-ligand 1; TMB, tumor mutational burden.

Supplementary Figure 2. Progression-free survival among the patients treated with gemcitabine plus platinum regimens [N=33]. All the 11 matched patients had at least one DNA-repair gene alteration in tissue DNA NGS (ID#4, #6, #12, #34, #35, #47, #66, #66, #67, #86, #99, and #118. **Supplementary Table 4**), including *BAP1* [N=4], *ATM* [N=2], *BRCA2* [N=2], *CHEK2* [N=1], *FANCL* [N=1], and *RAD50* [N=1].

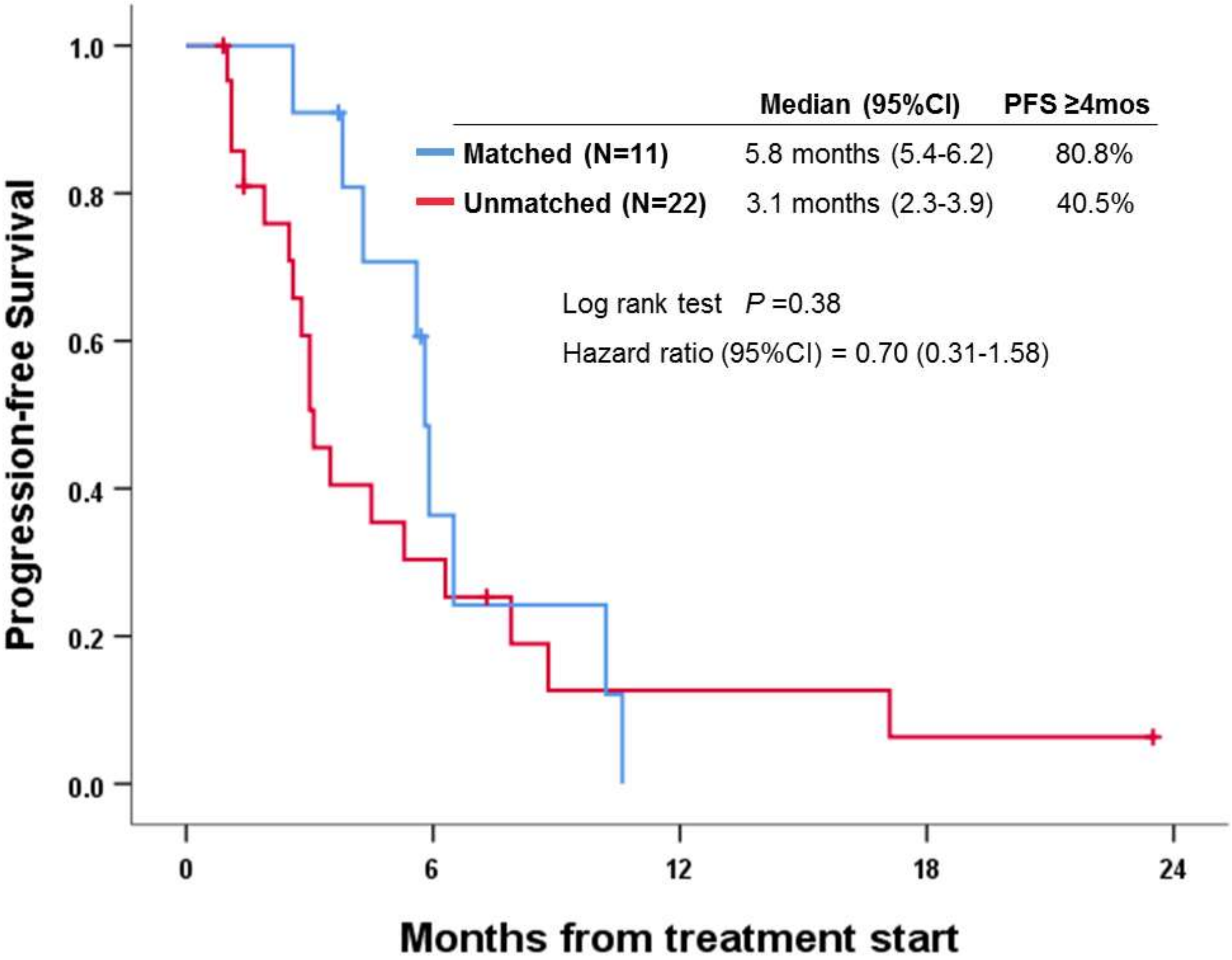

Supplementary Figure 3. Comparisons of treatment outcome according to matching score (>50% versus ≤50%).[88]
Panel 3A. Best response during the treatment (76 of 80 patients [95%] were available for RECIST evaluation). Among 76 evaluable patients, PR was observed in 10 patients (13%), SD in 25 (33%), and PD in 41 (54%) as the best response during the therapies following their molecular profiling.
Panel 3b. Progression-free survival (N=80).
Abbreviations: CI, confidence interval; PD, progressive disease; PFS, progression-free survival; PR, partial response.

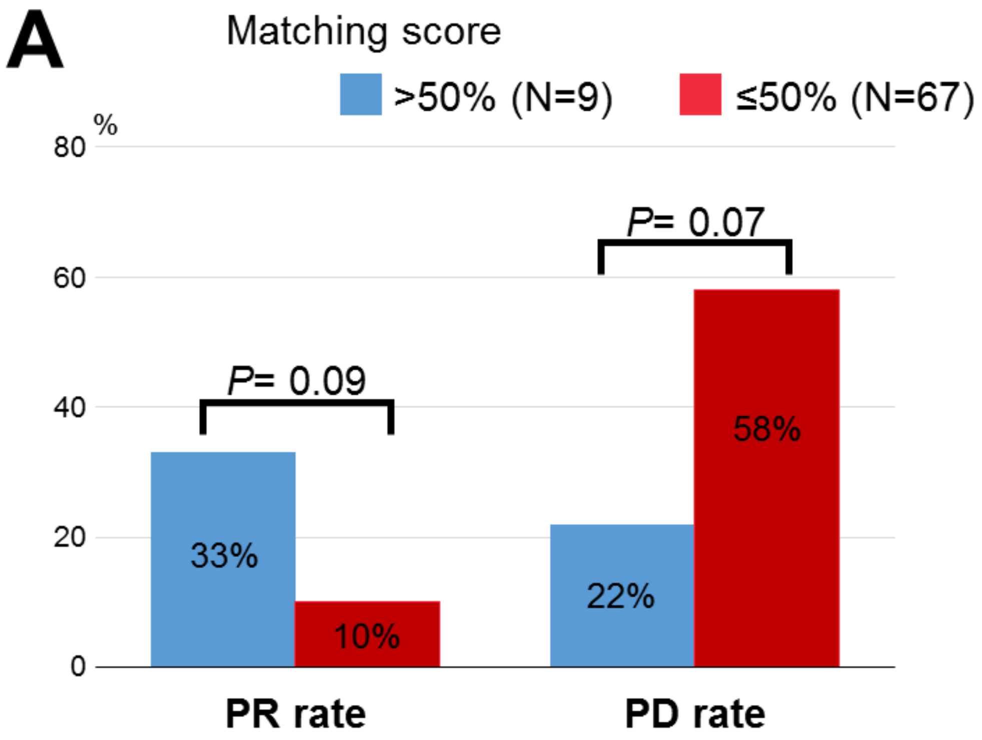

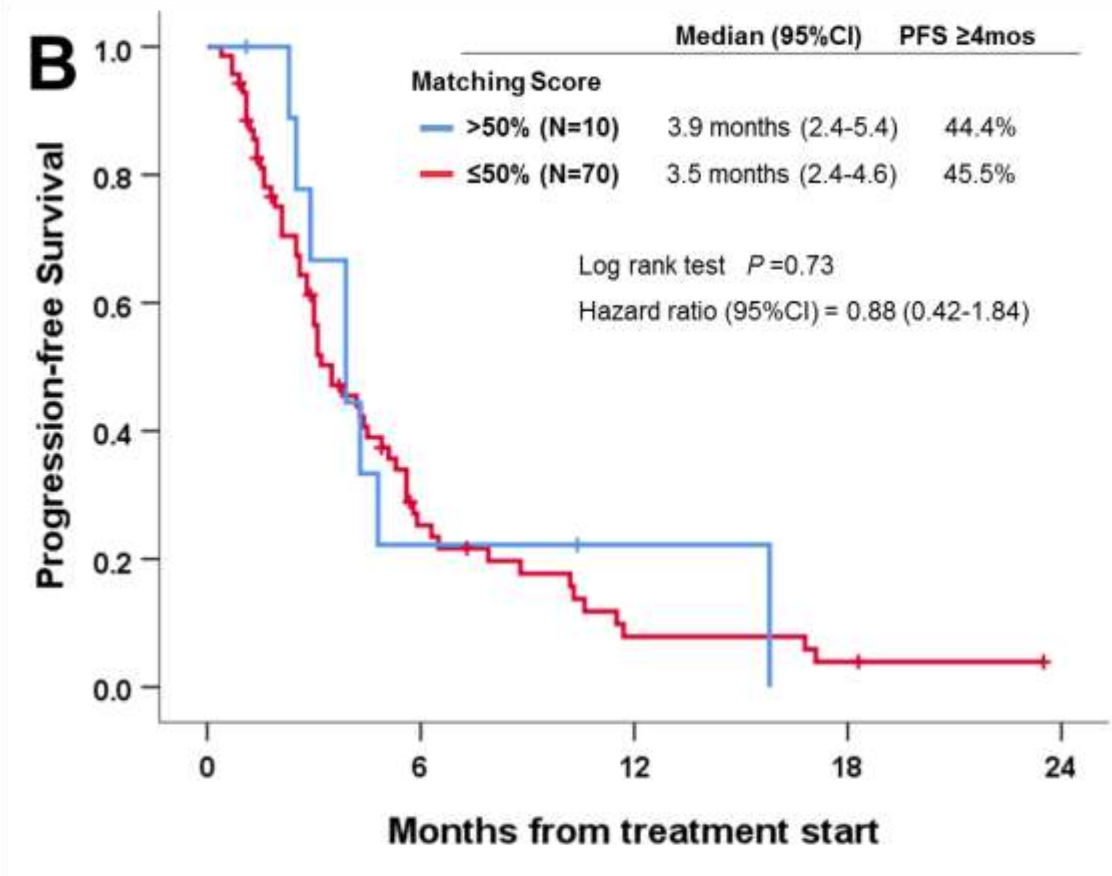

Supplementary Figure 4. Overall survival from the initiation of treatment in matched [N=34] versus unmatched [N=46].

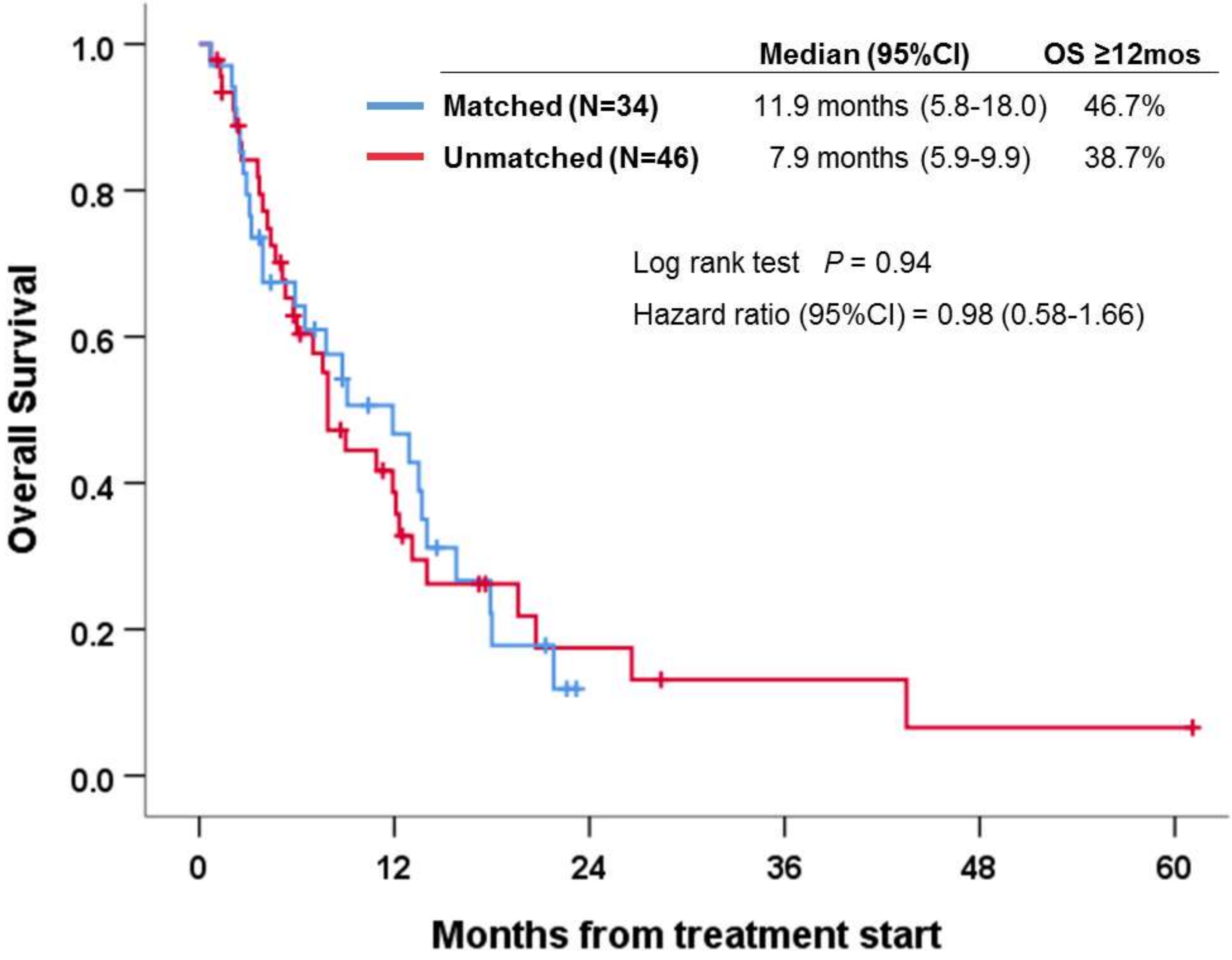

REFERENCES FOR SUPPLEMENTARY FILES

S1. Janku F, Wheler JJ, Westin SN, Moulder SL, Naing A, Tsimberidou AM, Fu S, Falchook GS, Hong DS, Garrido-Laguna I, Luthra R, Lee JJ, et al. PI3K/AKT/mTOR inhibitors in patients with breast and gynecologic malignancies harboring PIK3CA mutations. *Journal of clinical oncology : official journal of the American Society of Clinical Oncology* 2012;**30**: 777-82.

S2. Janku F, Hong DS, Fu S, Piha-Paul SA, Naing A, Falchook GS, Tsimberidou AM, Stepanek VM, Moulder SL, Lee JJ, Luthra R, Zinner RG, et al. Assessing PIK3CA and PTEN in early-phase trials with PI3K/AKT/mTOR inhibitors. *Cell reports* 2014;**6**: 377-87.

S3. Baselga J, Campone M, Piccart M, Burris HA, 3rd, Rugo HS, Sahmoud T, Noguchi S, Gnant M, Pritchard KI, Lebrun F, Beck JT, Ito Y, et al. Everolimus in postmenopausal hormone-receptor-positive advanced breast cancer. *The New England journal of medicine* 2012;**366**: 520-9.

S4. Kwitkowski VE, Prowell TM, Ibrahim A, Farrell AT, Justice R, Mitchell SS, Sridhara R, Pazdur R. FDA approval summary: temsirolimus as treatment for advanced renal cell carcinoma. *The oncologist* 2010;**15**: 428-35.

S5. Bertagnolli MM, Eagle CJ, Zauber AG, Redston M, Solomon SD, Kim K, Tang J, Rosenstein RB, Wittes J, Corle D, Hess TM, Woloj GM, et al. Celecoxib for the prevention of sporadic colorectal adenomas. *The New England journal of medicine* 2006;**355**: 873-84.

S6. Itano O, Yang K, Fan K, Kurihara N, Shinozaki H, Abe S, Jin B, Gravaghi C, Edelmann W, Augenlicht L, Kopelovich L, Kucherlapati R, et al. Sulindac effects on inflammation and tumorigenesis in the intestine of mice with Apc and Mlh1 mutations. *Carcinogenesis* 2009;**30**: 1923-6.

S7. Giardiello FM, Yang VW, Hylind LM, Krush AJ, Petersen GM, Trimbath JD, Piantadosi S, Garrett E, Geiman DE, Hubbard W, Offerhaus GJ, Hamilton SR. Primary chemoprevention of familial adenomatous polyposis with sulindac. *The New England journal of medicine* 2002;**346**: 1054-9.

S8. Patel JC, Maughan BL, Agarwal AM, Batten JA, Zhang TY, Agarwal N. Emerging molecularly targeted therapies in castration refractory prostate cancer. *Prostate cancer* 2013;**2013**: 981684.

S9. Chowdhury S, Kirby R. Advances in the treatment of metastatic prostate cancer. *The Practitioner* 2013;**257**: 15-8, 2.

S10. Imielinski M, Greulich H, Kaplan B, Araujo L, Amann J, Horn L, Schiller J, Villalona-Calero MA, Meyerson M, Carbone DP. Oncogenic and sorafenib-sensitive ARAF mutations in lung adenocarcinoma. *The Journal of clinical investigation* 2014;**124**: 1582-6.

S11. Michels J, Vitale I, Saparbaev M, Castedo M, Kroemer G. Predictive biomarkers for cancer therapy with PARP inhibitors. *Oncogene* 2014;**33**: 3894-907.

S12. Mateo J, Carreira S, Sandhu S, Miranda S, Mossop H, Perez-Lopez R, Nava Rodrigues D, Robinson D, Omlin A, Tunariu N, Boysen G, Porta N, et al. DNA-Repair Defects and Olaparib in Metastatic Prostate Cancer. *The New England journal of medicine* 2015;**373**: 1697-708.

S13. Bang YJ, Im SA, Lee KW, Cho JY, Song EK, Lee KH, Kim YH, Park JO, Chun HG, Zang DY, Fielding A, Rowbottom J, et al. Randomized, Double-Blind Phase II Trial With Prospective Classification by ATM Protein Level to Evaluate the Efficacy and Tolerability of Olaparib Plus Paclitaxel in Patients With Recurrent or Metastatic Gastric Cancer. *Journal of clinical oncology : official journal of the American Society of Clinical Oncology* 2015;**33**: 3858-65.

S14. Zhou L, Liu XD, Sun M, Zhang X, German P, Bai S, Ding Z, Tannir N, Wood CG, Matin SF, Karam JA, Tamboli P, et al. Targeting MET and AXL overcomes resistance to sunitinib therapy in renal cell carcinoma. *Oncogene* 2016;**35**: 2687-97.

S15. Pena-Llopis S, Vega-Rubin-de-Celis S, Liao A, Leng N, Pavia-Jimenez A, Wang S, Yamasaki T, Zhrebker L, Sivanand S, Spence P, Kinch L, Hambuch T, et al. BAP1 loss defines a new class of renal cell carcinoma. *Nature genetics* 2012;**44**: 751-9.

S16. Lord CJ, Ashworth A. BRCAness revisited. *Nature reviews Cancer* 2016;**16**: 110-20.

S17. Correia C, Schneider PA, Dai H, Dogan A, Maurer MJ, Church AK, Novak AJ, Feldman AL, Wu X, Ding H, Meng XW, Cerhan JR, et al. BCL2 mutations are associated with increased risk of transformation and shortened survival in follicular lymphoma. *Blood* 2015;**125**: 658-67.

S18. Tiacci E, Park JH, De Carolis L, Chung SS, Broccoli A, Scott S, Zaja F, Devlin S, Pulsoni A, Chung YR, Cimminiello M, Kim E, et al. Targeting Mutant BRAF in Relapsed or Refractory Hairy-Cell Leukemia. *The New England journal of medicine* 2015;**373**: 1733-47.

S19. Karoulia Z, Gavathiotis E, Poulikakos PI. New perspectives for targeting RAF kinase in human cancer. *Nature reviews Cancer* 2017;**17**: 676-91.

S20. Gray HJ, Bell-McGuinn K, Fleming GF, Cristea M, Xiong H, Sullivan D, Luo Y, McKee MD, Munasinghe W, Martin LP. Phase I combination study of the PARP inhibitor veliparib plus carboplatin and gemcitabine in patients with advanced ovarian cancer and other solid malignancies. *Gynecologic oncology* 2018;**148**: 507-14.

S21. Somlo G, Frankel PH, Arun BK, Ma CX, Garcia AA, Cigler T, Cream LV, Harvey HA, Sparano JA, Nanda R, Chew HK, Moynihan TJ, et al. Efficacy of the PARP Inhibitor Veliparib with Carboplatin or as a Single Agent in

Patients with Germline BRCA1- or BRCA2-Associated Metastatic Breast Cancer: California Cancer Consortium Trial NCT01149083. *Clinical cancer research : an official journal of the American Association for Cancer Research* 2017;**23**: 4066-76.
S22. DeMichele A, Clark AS, Tan KS, Heitjan DF, Gramlich K, Gallagher M, Lal P, Feldman M, Zhang P, Colameco C, Lewis D, Langer M, et al. CDK 4/6 inhibitor palbociclib (PD0332991) in Rb+ advanced breast cancer: phase II activity, safety, and predictive biomarker assessment. *Clinical cancer research : an official journal of the American Association for Cancer Research* 2015;**21**: 995-1001.
S23. Sherr CJ, Beach D, Shapiro GI. Targeting CDK4 and CDK6: From Discovery to Therapy. *Cancer discovery* 2016;**6**: 353-67.
S24. Gong X, Litchfield LM, Webster Y, Chio LC, Wong SS, Stewart TR, Dowless M, Dempsey J, Zeng Y, Torres R, Boehnke K, Mur C, et al. Genomic Aberrations that Activate D-type Cyclins Are Associated with Enhanced Sensitivity to the CDK4 and CDK6 Inhibitor Abemaciclib. *Cancer cell* 2017;**32**: 761-76.e6.
S25. Etemadmoghadam D, Weir BA, Au-Yeung G, Alsop K, Mitchell G, George J, Davis S, D'Andrea AD, Simpson K, Hahn WC, Bowtell DD. Synthetic lethality between CCNE1 amplification and loss of BRCA1. *Proceedings of the National Academy of Sciences of the United States of America* 2013;**110**: 19489-94.
S26. Dickson MA. Molecular pathways: CDK4 inhibitors for cancer therapy. *Clinical cancer research : an official journal of the American Association for Cancer Research* 2014;**20**: 3379-83.
S27. Finn RS, Crown JP, Lang I, Boer K, Bondarenko IM, Kulyk SO, Ettl J, Patel R, Pinter T, Schmidt M, Shparyk Y, Thummala AR, et al. The cyclin-dependent kinase 4/6 inhibitor palbociclib in combination with letrozole versus letrozole alone as first-line treatment of oestrogen receptor-positive, HER2-negative, advanced breast cancer (PALOMA-1/TRIO-18): a randomised phase 2 study. *The Lancet Oncology* 2015;**16**: 25-35.
S28. Lachenmayer A, Alsinet C, Savic R, Cabellos L, Toffanin S, Hoshida Y, Villanueva A, Minguez B, Newell P, Tsai HW, Barretina J, Thung S, et al. Wnt-pathway activation in two molecular classes of hepatocellular carcinoma and experimental modulation by sorafenib. *Clinical cancer research : an official journal of the American Association for Cancer Research* 2012;**18**: 4997-5007.
S29. Metzeler KH, Walker A, Geyer S, Garzon R, Klisovic RB, Bloomfield CD, Blum W, Marcucci G. DNMT3A mutations and response to the hypomethylating agent decitabine in acute myeloid leukemia. *Leukemia* 2012;**26**: 1106-7.
S30. Baselga J. The EGFR as a target for anticancer therapy--focus on cetuximab. *European journal of cancer (Oxford, England : 1990)* 2001;**37 Suppl 4**: S16-22.
S31. Sequist LV, Yang JC, Yamamoto N, O'Byrne K, Hirsh V, Mok T, Geater SL, Orlov S, Tsai CM, Boyer M, Su WC, Bennouna J, et al. Phase III study of afatinib or cisplatin plus pemetrexed in patients with metastatic lung adenocarcinoma with EGFR mutations. *Journal of clinical oncology : official journal of the American Society of Clinical Oncology* 2013;**31**: 3327-34.
S32. Zhou C, Wu YL, Chen G, Feng J, Liu XQ, Wang C, Zhang S, Wang J, Zhou S, Ren S, Lu S, Zhang L, et al. Erlotinib versus chemotherapy as first-line treatment for patients with advanced EGFR mutation-positive non-small-cell lung cancer (OPTIMAL, CTONG-0802): a multicentre, open-label, randomised, phase 3 study. *The Lancet Oncology* 2011;**12**: 735-42.
S33. Richard S, Selle F, Lotz JP, Khalil A, Gligorov J, Soares DG. Pertuzumab and trastuzumab: the rationale way to synergy. *Anais da Academia Brasileira de Ciencias* 2016;**88 Suppl 1**: 565-77.
S34. Fuentes G, Scaltriti M, Baselga J, Verma CS. Synergy between trastuzumab and pertuzumab for human epidermal growth factor 2 (Her2) from colocalization: an in silico based mechanism. *Breast cancer research : BCR* 2011;**13**: R54.
S35. Esteva FJ, Pusztai L. Optimizing outcomes in HER2-positive breast cancer: the molecular rationale. *Oncology (Williston Park, NY)* 2005;**19**: 5-16.
S36. Hainsworth JD, Meric-Bernstam F, Swanton C, Hurwitz H, Spigel DR, Sweeney C, Burris H, Bose R, Yoo B, Stein A, Beattie M, Kurzrock R. Targeted Therapy for Advanced Solid Tumors on the Basis of Molecular Profiles: Results From MyPathway, an Open-Label, Phase IIa Multiple Basket Study. *Journal of clinical oncology : official journal of the American Society of Clinical Oncology* 2018;**36**: 536-42.
S37. Modjtahedi H, Cho BC, Michel MC, Solca F. A comprehensive review of the preclinical efficacy profile of the ErbB family blocker afatinib in cancer. *Naunyn-Schmiedeberg's archives of pharmacology* 2014;**387**: 505-21.
S38. Toy W, Weir H, Razavi P, Lawson M, Goeppert AU, Mazzola AM, Smith A, Wilson J, Morrow C, Wong WL, De Stanchina E, Carlson KE, et al. Activating ESR1 Mutations Differentially Affect the Efficacy of ER Antagonists. *Cancer discovery* 2017;**7**: 277-87.
S39. Mao JH, Kim IJ, Wu D, Climent J, Kang HC, DelRosario R, Balmain A. FBXW7 targets mTOR for degradation and cooperates with PTEN in tumor suppression. *Science (New York, NY)* 2008;**321**: 1499-502.
S40. Jardim DL, Wheler JJ, Hess K, Tsimberidou AM, Zinner R, Janku F, Subbiah V, Naing A, Piha-Paul SA, Westin SN, Roy-Chowdhuri S, Meric-Bernstam F, et al. FBXW7 mutations in patients with advanced cancers: clinical and molecular characteristics and outcomes with mTOR inhibitors. *PloS one* 2014;**9**: e89388.

S41. Myers AP, Filiaci VL, Zhang Y, Pearl M, Behbakht K, Makker V, Hanjani P, Zweizig S, Burke JJ, 2nd, Downey G, Leslie KK, Van Hummelen P, et al. Tumor mutational analysis of GOG248, a phase II study of temsirolimus or temsirolimus and alternating megestrol acetate and tamoxifen for advanced endometrial cancer (EC): An NRG Oncology/Gynecologic Oncology Group study. *Gynecologic oncology* 2016;**141**: 43-8.
S42. Matsuki M, Hoshi T, Yamamoto Y, Ikemori-Kawada M, Minoshima Y, Funahashi Y, Matsui J. Lenvatinib inhibits angiogenesis and tumor fibroblast growth factor signaling pathways in human hepatocellular carcinoma models. *Cancer medicine* 2018;**7**: 2641-53.
S43. Tohyama O, Matsui J, Kodama K, Hata-Sugi N, Kimura T, Okamoto K, Minoshima Y, Iwata M, Funahashi Y. Antitumor activity of lenvatinib (e7080): an angiogenesis inhibitor that targets multiple receptor tyrosine kinases in preclinical human thyroid cancer models. *Journal of thyroid research* 2014;**2014**: 638747.
S44. Moreira RB, Peixoto RD, de Sousa Cruz MR. Clinical Response to Sorafenib in a Patient with Metastatic Colorectal Cancer and FLT3 Amplification. *Case reports in oncology* 2015;**8**: 83-7.
S45. Gozgit JM, Wong MJ, Wardwell S, Tyner JW, Loriaux MM, Mohemmad QK, Narasimhan NI, Shakespeare WC, Wang F, Druker BJ, Clackson T, Rivera VM. Potent activity of ponatinib (AP24534) in models of FLT3-driven acute myeloid leukemia and other hematologic malignancies. *Molecular cancer therapeutics* 2011;**10**: 1028-35.
S46. O'Hare T, Shakespeare WC, Zhu X, Eide CA, Rivera VM, Wang F, Adrian LT, Zhou T, Huang WS, Xu Q, Metcalf CA, 3rd, Tyner JW, et al. AP24534, a pan-BCR-ABL inhibitor for chronic myeloid leukemia, potently inhibits the T315I mutant and overcomes mutation-based resistance. *Cancer cell* 2009;**16**: 401-12.
S47. Fiedler W, Kayser S, Kebenko M, Janning M, Krauter J, Schittenhelm M, Gotze K, Weber D, Gohring G, Teleanu V, Thol F, Heuser M, et al. A phase I/II study of sunitinib and intensive chemotherapy in patients over 60 years of age with acute myeloid leukaemia and activating FLT3 mutations. *British journal of haematology* 2015;**169**: 694-700.
S48. Baker SD, Zimmerman EI, Wang YD, Orwick S, Zatechka DS, Buaboonnam J, Neale GA, Olsen SR, Enemark EJ, Shurtleff S, Rubnitz JE, Mullighan CG, et al. Emergence of polyclonal FLT3 tyrosine kinase domain mutations during sequential therapy with sorafenib and sunitinib in FLT3-ITD-positive acute myeloid leukemia. *Clinical cancer research : an official journal of the American Association for Cancer Research* 2013;**19**: 5758-68.
S49. Schechter RB, Nagilla M, Joseph L, Reddy P, Khattri A, Watson S, Locati LD, Licitra L, Greco A, Pelosi G, Carcangiu ML, Lingen MW, et al. Genetic profiling of advanced radioactive iodine-resistant differentiated thyroid cancer and correlation with axitinib efficacy. *Cancer letters* 2015;**359**: 269-74.
S50. Zhang K, Chu K, Wu X, Gao H, Wang J, Yuan YC, Loera S, Ho K, Wang Y, Chow W, Un F, Chu P, et al. Amplification of FRS2 and activation of FGFR/FRS2 signaling pathway in high-grade liposarcoma. *Cancer research* 2013;**73**: 1298-307.
S51. Cox AD, Fesik SW, Kimmelman AC, Luo J, Der CJ. Drugging the undruggable RAS: Mission possible? *Nature reviews Drug discovery* 2014;**13**: 828-51.
S52. Ideno N, Yamaguchi H, Ghosh B, Gupta S, Okumura T, Steffen DJ, Fisher CG, Wood LD, Singhi AD, Nakamura M, Gutkind JS, Maitra A. GNAS(R201C) Induces Pancreatic Cystic Neoplasms in Mice That Express Activated KRAS by Inhibiting YAP1 Signaling. *Gastroenterology* 2018;**155**: 1593-607.e12.
S53. Flaherty KT, Robert C, Hersey P, Nathan P, Garbe C, Milhem M, Demidov LV, Hassel JC, Rutkowski P, Mohr P, Dummer R, Trefzer U, et al. Improved survival with MEK inhibition in BRAF-mutated melanoma. *The New England journal of medicine* 2012;**367**: 107-14.
S54. Larkin J, Ascierto PA, Dreno B, Atkinson V, Liszkay G, Maio M, Mandala M, Demidov L, Stroyakovskiy D, Thomas L, de la Cruz-Merino L, Dutriaux C, et al. Combined vemurafenib and cobimetinib in BRAF-mutated melanoma. *The New England journal of medicine* 2014;**371**: 1867-76.
S55. Yakes FM, Chen J, Tan J, Yamaguchi K, Shi Y, Yu P, Qian F, Chu F, Bentzien F, Cancilla B, Orf J, You A, et al. Cabozantinib (XL184), a novel MET and VEGFR2 inhibitor, simultaneously suppresses metastasis, angiogenesis, and tumor growth. *Molecular cancer therapeutics* 2011;**10**: 2298-308.
S56. DiNardo CD, Stein EM, de Botton S, Roboz GJ, Altman JK, Mims AS, Swords R, Collins RH, Mannis GN, Pollyea DA, Donnellan W, Fathi AT, et al. Durable Remissions with Ivosidenib in IDH1-Mutated Relapsed or Refractory AML. *The New England journal of medicine* 2018;**378**: 2386-98.
S57. Rohle D, Popovici-Muller J, Palaskas N, Turcan S, Grommes C, Campos C, Tsoi J, Clark O, Oldrini B, Komisopoulou E, Kunii K, Pedraza A, et al. An inhibitor of mutant IDH1 delays growth and promotes differentiation of glioma cells. *Science (New York, NY)* 2013;**340**: 626-30.
S58. Harry BL, Eckhardt SG, Jimeno A. JAK2 inhibition for the treatment of hematologic and solid malignancies. *Expert opinion on investigational drugs* 2012;**21**: 637-55.
S59. Janne PA, van den Heuvel MM, Barlesi F, Cobo M, Mazieres J, Crino L, Orlov S, Blackhall F, Wolf J, Garrido P, Poltoratskiy A, Mariani G, et al. Selumetinib Plus Docetaxel Compared With Docetaxel Alone and Progression-Free Survival in Patients With KRAS-Mutant Advanced Non-Small Cell Lung Cancer: The SELECT-1 Randomized Clinical Trial. *Jama* 2017;**317**: 1844-53.

S60. Adjei AA, Cohen RB, Franklin W, Morris C, Wilson D, Molina JR, Hanson LJ, Gore L, Chow L, Leong S, Maloney L, Gordon G, et al. Phase I pharmacokinetic and pharmacodynamic study of the oral, small-molecule mitogen-activated protein kinase kinase 1/2 inhibitor AZD6244 (ARRY-142886) in patients with advanced cancers. *Journal of clinical oncology : official journal of the American Society of Clinical Oncology* 2008;**26**: 2139-46.
S61. Manchado E, Weissmueller S, Morris JPt, Chen CC, Wullenkord R, Lujambio A, de Stanchina E, Poirier JT, Gainor JF, Corcoran RB, Engelman JA, Rudin CM, et al. A combinatorial strategy for treating KRAS-mutant lung cancer. *Nature* 2016;**534**: 647-51.
S62. Blumenschein GR, Jr., Smit EF, Planchard D, Kim DW, Cadranel J, De Pas T, Dunphy F, Udud K, Ahn MJ, Hanna NH, Kim JH, Mazieres J, et al. A randomized phase II study of the MEK1/MEK2 inhibitor trametinib (GSK1120212) compared with docetaxel in KRAS-mutant advanced non-small-cell lung cancer (NSCLC)dagger. *Annals of oncology : official journal of the European Society for Medical Oncology* 2015;**26**: 894-901.
S63. Zhu Z, Aref AR, Cohoon TJ, Barbie TU, Imamura Y, Yang S, Moody SE, Shen RR, Schinzel AC, Thai TC, Reibel JB, Tamayo P, et al. Inhibition of KRAS-driven tumorigenicity by interruption of an autocrine cytokine circuit. *Cancer discovery* 2014;**4**: 452-65.
S64. Falchook GS, Lewis KD, Infante JR, Gordon MS, Vogelzang NJ, DeMarini DJ, Sun P, Moy C, Szabo SA, Roadcap LT, Peddareddigari VG, Lebowitz PF, et al. Activity of the oral MEK inhibitor trametinib in patients with advanced melanoma: a phase 1 dose-escalation trial. *The Lancet Oncology* 2012;**13**: 782-9.
S65. Garber K. MET inhibitors start on road to recovery. *Nature reviews Drug discovery* 2014;**13**: 563-5.
S66. Le DT, Durham JN, Smith KN, Wang H, Bartlett BR, Aulakh LK, Lu S, Kemberling H, Wilt C, Luber BS, Wong F, Azad NS, et al. Mismatch repair deficiency predicts response of solid tumors to PD-1 blockade. *Science (New York, NY)* 2017;**357**: 409-13.
S67. Le DT, Uram JN, Wang H, Bartlett BR, Kemberling H, Eyring AD, Skora AD, Luber BS, Azad NS, Laheru D, Biedrzycki B, Donehower RC, et al. PD-1 Blockade in Tumors with Mismatch-Repair Deficiency. *The New England journal of medicine* 2015;**372**: 2509-20.
S68. Lodish MB, Stratakis CA. Endocrine tumours in neurofibromatosis type 1, tuberous sclerosis and related syndromes. *Best practice & research Clinical endocrinology & metabolism* 2010;**24**: 439-49.
S69. Hattori S, Ohmi N, Maekawa M, Hoshino M, Kawakita M, Nakamura S. Antibody against neurofibromatosis type 1 gene product reacts with a triton-insoluble GTPase activating protein toward ras p21. *Biochemical and biophysical research communications* 1991;**177**: 83-9.
S70. Schroeder RD, Angelo LS, Kurzrock R. NF2/merlin in hereditary neurofibromatosis 2 versus cancer: biologic mechanisms and clinical associations. *Oncotarget* 2014;**5**: 67-77.
S71. Taube JM, Klein A, Brahmer JR, Xu H, Pan X, Kim JH, Chen L, Pardoll DM, Topalian SL, Anders RA. Association of PD-1, PD-1 ligands, and other features of the tumor immune microenvironment with response to anti-PD-1 therapy. *Clinical cancer research : an official journal of the American Association for Cancer Research* 2014;**20**: 5064-74.
S72. Topalian SL, Hodi FS, Brahmer JR, Gettinger SN, Smith DC, McDermott DF, Powderly JD, Carvajal RD, Sosman JA, Atkins MB, Leming PD, Spigel DR, et al. Safety, activity, and immune correlates of anti-PD-1 antibody in cancer. *The New England journal of medicine* 2012;**366**: 2443-54.
S73. Heldin CH. Targeting the PDGF signaling pathway in tumor treatment. *Cell communication and signaling : CCS* 2013;**11**: 97.
S74. Cheung LW, Hennessy BT, Li J, Yu S, Myers AP, Djordjevic B, Lu Y, Stemke-Hale K, Dyer MD, Zhang F, Ju Z, Cantley LC, et al. High frequency of PIK3R1 and PIK3R2 mutations in endometrial cancer elucidates a novel mechanism for regulation of PTEN protein stability. *Cancer discovery* 2011;**1**: 170-85.
S75. LoRusso PM, Rudin CM, Reddy JC, Tibes R, Weiss GJ, Borad MJ, Hann CL, Brahmer JR, Chang I, Darbonne WC, Graham RA, Zerivitz KL, et al. Phase I trial of hedgehog pathway inhibitor vismodegib (GDC-0449) in patients with refractory, locally advanced or metastatic solid tumors. *Clinical cancer research : an official journal of the American Association for Cancer Research* 2011;**17**: 2502-11.
S76. Wu R, Hu TC, Rehemtulla A, Fearon ER, Cho KR. Preclinical testing of PI3K/AKT/mTOR signaling inhibitors in a mouse model of ovarian endometrioid adenocarcinoma. *Clinical cancer research : an official journal of the American Association for Cancer Research* 2011;**17**: 7359-72.
S77. Kuusisto KM, Bebel A, Vihinen M, Schleutker J, Sallinen SL. Screening for BRCA1, BRCA2, CHEK2, PALB2, BRIP1, RAD50, and CDH1 mutations in high-risk Finnish BRCA1/2-founder mutation-negative breast and/or ovarian cancer individuals. *Breast cancer research : BCR* 2011;**13**: R20.
S78. Abuzeid WM, Jiang X, Shi G, Wang H, Paulson D, Araki K, Jungreis D, Carney J, O'Malley BW, Jr., Li D. Molecular disruption of RAD50 sensitizes human tumor cells to cisplatin-based chemotherapy. *The Journal of clinical investigation* 2009;**119**: 1974-85.
S79. Figures MR, Wobb J, Araki K, Liu T, Xu L, Zhu H, O'Malley BW, Jr., Li D. Head and neck squamous cell carcinoma targeted chemosensitization. *Otolaryngology--head and neck surgery : official journal of American Academy of Otolaryngology-Head and Neck Surgery* 2009;**141**: 177-83.

S80. Montero JC, Seoane S, Ocana A, Pandiella A. Inhibition of SRC family kinases and receptor tyrosine kinases by dasatinib: possible combinations in solid tumors. *Clinical cancer research : an official journal of the American Association for Cancer Research* 2011;**17**: 5546-52.
S81. Dancey J. mTOR signaling and drug development in cancer. *Nature reviews Clinical oncology* 2010;**7**: 209-19.
S82. Klumpen HJ, Queiroz KC, Spek CA, van Noesel CJ, Brink HC, de Leng WW, de Wilde RF, Mathus-Vliegen EM, Offerhaus GJ, Alleman MA, Westermann AM, Richel DJ. mTOR inhibitor treatment of pancreatic cancer in a patient With Peutz-Jeghers syndrome. *Journal of clinical oncology : official journal of the American Society of Clinical Oncology* 2011;**29**: e150-3.
S83. Schwaederle M, Lazar V, Validire P, Hansson J, Lacroix L, Soria JC, Pawitan Y, Kurzrock R. VEGF-A Expression Correlates with TP53 Mutations in Non-Small Cell Lung Cancer: Implications for Antiangiogenesis Therapy. *Cancer research* 2015;**75**: 1187-90.
S84. Koehler K, Liebner D, Chen JL. TP53 mutational status is predictive of pazopanib response in advanced sarcomas. *Annals of oncology : official journal of the European Society for Medical Oncology* 2016;**27**: 539-43.
S85. Wheler JJ, Janku F, Naing A, Li Y, Stephen B, Zinner R, Subbiah V, Fu S, Karp D, Falchook GS, Tsimberidou AM, Piha-Paul S, et al. TP53 Alterations Correlate with Response to VEGF/VEGFR Inhibitors: Implications for Targeted Therapeutics. *Molecular cancer therapeutics* 2016;**15**: 2475-85.
S86. Li AM, Boichard A, Kurzrock R. Mutated TP53 is a marker of increased VEGF expression: analysis of 7,525 pan-cancer tissues. *Cancer biology & therapy* 2019: 1-6.
S87. Ferrara N, Hillan KJ, Novotny W. Bevacizumab (Avastin), a humanized anti-VEGF monoclonal antibody for cancer therapy. *Biochemical and biophysical research communications* 2005;**333**: 328-35.
S88. Sicklick JK, Kato S, Okamura R, Schwaederle M, Hahn ME, Williams CB, De P, Krie A, Piccioni DE, Miller VA, Ross JS, Benson A, et al. Molecular profiling of cancer patients enables personalized combination therapy: the I-PREDICT study. *Nature medicine* 2019.
